# Scoping review of epigenetics on neurodegenerative diseases: research frontiers and publication status

**DOI:** 10.3389/fnins.2024.1414603

**Published:** 2024-10-09

**Authors:** Yanyan Zhang, Yukang Mao, Qiangqiang Fu, Xiaoguang Zhang, Dong Zhang, Yunhua Yue, Chuanxi Yang

**Affiliations:** ^1^Department of Neurology, Yangpu Hospital, School of Medicine, Tongji University, Shanghai, China; ^2^Department of Cardiology, The Affiliated Suzhou Hospital of Nanjing Medical University, Suzhou Municipal Hospital, Gusu School, Nanjing Medical University, Suzhou, Jiangsu, China; ^3^Department of Cardiology, The First Affiliated Hospital of Nanjing Medical University, Nanjing, Jiangsu, China; ^4^Department of General Practice, Clinical Research Center for General Practice, Yangpu Hospital, Tongji University School of Medicine, Shanghai, China; ^5^Department of Cardiology, Yangpu Hospital, Tongji University School of Medicine, Shanghai, China; ^6^Center for Clinical Research and Translational Medicine, Yangpu Hospital, Tongji University School of Medicine, Shanghai, China

**Keywords:** scientometric, neurodegenerative diseases, epigenetics, evidence synthesis, hotspots

## Abstract

**Aims:**

Epigenetics has significantly evolved and emerged as important players in the pathogenesis of neurodegenerative diseases. However, a scientometric synthesis of such changes over time is currently lacking.

**Methods:**

We conducted a comprehensive search of the Web of Science Core Collection from inception until November 5, 2022, using appropriate keywords. Our primary objective was to employ scientometric analysis to depict changes in keywords over time and to assess the structure and credibility of clusters. Additionally, we examined the network of research (countries, institutions, and authors) using CiteSpace and VOSviewer.

**Results:**

We identified 25 clusters with well-structured networks (*Q* = 0.82) and highly credible clustering (*S* = 0.91) from 16,181 articles published between 1999 and 2022. Our findings are as follows: (a) the literature and research interest concerning the epigenetics of neurodegenerative diseases are continuously growing; (b) the three most productive countries are the USA, China, and Germany; (c) international collaborative relationships exist, alongside small, isolated collaboration networks of individual institutions.

**Conclusion:**

The number and impact of global publications on the epigenetics of neurodegenerative diseases have expanded rapidly over the past 20 years. This review provides valuable guidelines for researchers interested in neurodegenerative diseases research.

## Introduction

Neurodegenerative diseases (NDs) are characterized by the loss and dysfunction of the nervous system, posing a significant global health burden. Alzheimer’s disease (AD), Parkinson’s disease (PD), and amyotrophic lateral sclerosis (ALS) represent major types of NDs (Berson et al., 2018). The etiology of NDs involves aging as a primary factor, along with genetic mutations and various environmental influences, such as neuroinflammation, mitochondrial dysfunction, cell apoptosis, and oxidative stress. Understanding the molecular mechanisms underlying these pathological changes is crucial for developing effective therapeutic strategies. However, the relationship between genetics and external risk factors in NDs remains a subject of debate. Nonetheless, emerging evidence from clinical and experimental studies suggests the potential for timely diagnostic markers and therapeutic interventions to prevent or slow the progression of NDs. Given the increasing incidence of these diseases, there is a pressing need for the development of novel therapeutic strategies, elucidation of disease mechanisms, identification of reliable biomarkers, and establishment of therapeutic targets.

The interplay between genetics and the environment in NDs was highlighted by Conrad Waddington in 1940 when he proposed epigenetics (Nakagawa et al., 2019). Epigenetics elucidates the complex relationship between environmental factors and genetics, demonstrating heritable changes in gene expression without alterations in DNA sequence. DNA methylation, the first recognized epigenetic modification, regulates gene expression, and subsequent research has identified additional modifications, such as hydroxymethylation, carboxylation, and histone post-translational modifications. Epigenetic mechanisms, including RNA interference and chromatin modifications, play pivotal roles in regulating gene expression, cell type-specific gene expression, imprinting, and heterochromatin silencing. Studies have shown that epigenetic modifications, including DNA methylation, RNA interference, and chromatin modifications, contribute to the development of chronic diseases such as diabetes, hypertension, and NDs. Given the multifactorial nature of NDs, advances in epigenetics research hold promise for elucidating etiological and pathogenetic processes and identifying targets for early diagnosis and therapeutic intervention.

While considerable research has been conducted on the epigenomics of NDs over the past three decades, few studies have provided a comprehensive understanding of the field. In 2019, Nakagawa et al. introduced the “research weaving” framework, which integrates evidence synthesis and influence analysis. By combining systematic mapping and bibliometrics, research weaving offers a comprehensive overview of a research field, identifying areas requiring further investigation and synthesizing existing evidence. Despite the wealth of research on NDs and epigenetics, no scientometric study has systematically analyzed publication trends in this area. Bibliometrics (termed science of literature) has devolved into an important research concept since its first proposal in 1969, in which a series of bibliometric indexes, such as the amount and impact of publications, contributions of countries, institutions or authors, cooperation between different countries, institutions or authors were comprehensively assessed to depict the developing landscape of a study theme (Nakagawa et al., 2019). Recently, with the advent of the information era, several analytical software tools—specifically Bibliometrics, VOSviewer, and CiteSapce—were designed and widely applied in processing big data and visualizing bibliometric information, facilitating the accurate identification of the up-to-date research trends and hotspot topics in a field of interest and the prediction of the frontiers and future directions. Therefore, in this paper, we conducted bibliometric analyses and systematic mapping to explore the epigenetics of NDs.

## Materials and methods

### Objectives

Our main objective is to conduct a comprehensive systematic mapping of genetic studies related to neurodegenerative diseases, aiming to elucidate temporal trends in these diseases and to identify key research themes utilizing co-citation reference networks and keywords. A secondary objective is to provide research network metrics (national, institutional, authorial, and periodical) for clinicians and researchers, enabling the identification of research response relationships, disparities, emerging trends, biases, and limitations.

### Search policy and data collection

Researchers employed a comprehensive search strategy encompassing various terms and phrases associated with the genetics of neurodegenerative diseases. Medical subject headings and keywords relevant to the research scope were utilized to ensure thorough coverage (see Supplementary Information 1). The analysis exclusively considers documents classified as “articles” or “comments, excluding reviews, letters, editorials, and guidelines. There were no restrictions based on language or publication date. The bibliography includes records and references published up to November 19, 2022, with duplicate entries removed using CiteSpace.

### Data analysis

Following data extraction, analysis was conducted using the Bibliometrix R package (version 3.1.4), VOSviewer (version 1.6.17), and CiteSpace (version 5.8.R3). Various bibliographic indicators, such as authors, journals, references, countries, institutions, and keywords, were examined. The Bibliometrix R package, utilized with R version 4.0.5, provided information on authors and journals. VOSviewer generated network maps illustrating the most-cited journals and co-occurring author keywords. CiteSpace facilitated the extraction of data and the visualization of collaboration networks across countries and institutions, co-citation analyses, and co-occurrence analyses of author keywords. Burst analyses were conducted for all units of measure. The modularity score (also termed the Q score), for which the theoretical range is 0–1, was leverged as an indicator of whether a cluster can be explicitly distinguished in the network. A network would only be deemed significantly structured when Q score is calculated to exceed 0.3 (Newman, 2006). The silhouette score (also termed the S score) (ranging from −1 to 1), helps to assess the robustness of results garnered from clustering analysis of a network. An S score higher than 0.3, 0.5, and 0.7 is recognized as the major criteria for determining the homogeneity, reasonability, and credibility of a network, respectively (Rousseeuw, 1987). The g-index, an author-level metric accounting for both highly cited and less cited papers, was employed for calculations. Notably, the g-index is particularly effective in assessing papers with low or no citations, thereby enhancing the accuracy of citation analyses. CiteSpace also optimized time slices by removing blank intervals and refining the time range. Details of CiteSpace parameters are provided in Supplementary Figure 1.

## Results

### Analysis of co-cited references: clusters of research and most cited papers

#### Clusters of research

**FIGURE 1:**
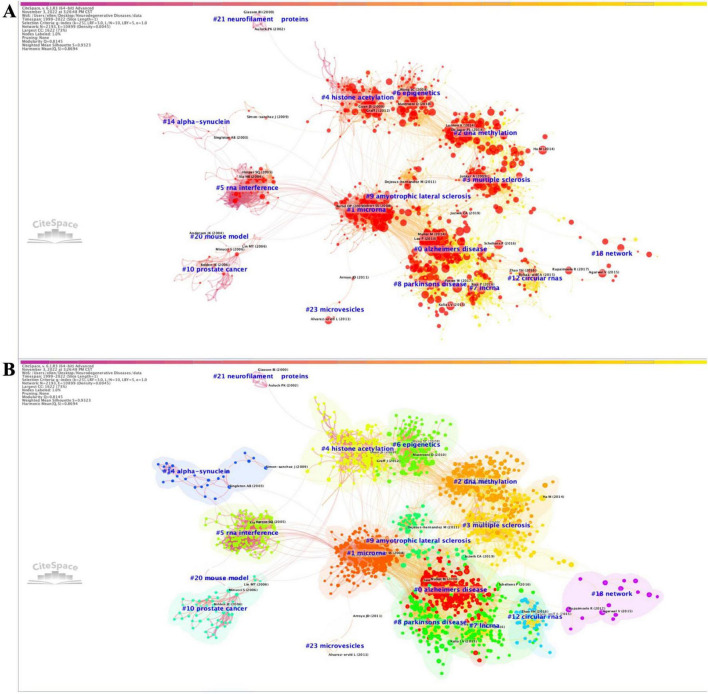
Co-citation references network (1999–2022) and correspondent clustering analysis obtained with CiteSpace. **(A)** Co-citation reference network with cluster visualization and burstness of hotspots. **(B)** Visualization map of the corresponding clusters and burestness of hotspots. The size of a node (article) is proportional to the number of times the article has been co-cited. Burstness is represented by red tree rings, with either important citation burst.

Utilizing the software CiteSpace, we generated a network of reference co-citations with cluster visualization and identified bursts of hotspots among landmark references (Figures 1A, B). As depicted in Figure 1, our analysis of co-cited references revealed significant modularity and silhouette scores, indicating highly credible clusters (Q = 0.8145; S = 0.9323). We identified 25 distinct clusters, the details of which are available in Supplementary Figure 2 and Supplementary Table 1.

From the figures and tables presented, we observed two major trends in research regrouped within a single constellation. The first trend focused on the expression control of posttranscriptional genes, initiating between 2000 and 2010 with two research clusters: clusters #14 (“alpha-synuclein”; 29; S = 0.988; 2003) (Xu and Hebert, 2005) and #20 (“mouse model”; 6; S = 0.984; 2004) (Richard et al., 2007). These clusters shared hotspots with cluster #5 (“RNA interference”; 141; S = 0.934; 2003) (Thakker et al., 2006) and #1 (“microRNA”; 209; S = 0.9; 2008) (Lardenoije et al., 2015), which were further studied in clusters #0 (“Alzheimer’s disease”; 236; S = 0.932; 2016) (Müller et al., 2014), #8 (“Parkinson’s disease”; 93; S = 0.932; 2015) (Leggio et al., 2017), and #9 (“amyotrophic lateral sclerosis”; 69; S = 0.942; 2013) (DeJesus-Hernandez et al., 2011). Subsequently, these clusters evolved into clusters #7 (“lncRNA”; 110; S = 0.915; 2017) (Zhang et al., 2021) and #12 (“circular RNAs”; 42; S = 0.988; 2016) (Salvatori et al., 2020).

The second major trend of research focused on the regulation of selective gene transcription expression in neurodegenerative diseases (NDs). It commenced with research on cluster #4 (“histone acetylation”; 144; S = 0.965; 2007), which further developed into clusters #6 (“epigenetics”; 125; S = 0.918; 2010) and #2 (“DNA methylation”; 187; S = 0.932; 2015) (De Jager et al., 2014; Lunnon et al., 2014), concerning effects in cluster #3 (“multiple sclerosis”; 151; S = 0.942; 2012) (Junker et al., 2009). Additionally, three relatively independent trends of research were identified: one on #21 (“neurofilament proteins”; 6; S = 0.999; 2000) (Giasson et al., 2000), one on #10 (“prostate cancer”; 50; S = 0.993; 2002) (Bolden et al., 2006), and one on #23 (“microvesicles”; 5; S = 0.997; 2009) (Alvarez-Erviti et al., 2011a).

Based on the burstness dynamics of this co-cited reference network (1999–2022), the link walkthrough over time between clusters is available in Supplementary Figure 3. To further explore research trends, we focused on the co-citation reference network of the last 5 years (2017–2022) and each month of the last available year (2022) (Supplementary Figures 4–6). For both networks (Supplementary Figures 4, 6), the modularity score was significant, and the silhouette suggested highly credible clusters (Q = 0.7277; S = 0.9038 and Q = 0.6818; S = 0.8798, respectively). The two corresponding networks revealed the evolution of two major research trends previously identified in Figure 1. “Alzheimer’s disease” became a cluster with methylation studies #6 (“DNA methylation”; 65; S = 0.836; 2019) (Wang et al., 2018) and #2 (“miR-132”; 83; S = 0.856; 2015) (Juźwik et al., 2019), further evolving into clusters #3 (“heavy metals”; 38; S = 0.852; 2019) (Chen et al., 2020), #5 (“circular RNAs”; 37; S = 0.876; 2018) (Cha et al., 2019), and #6 (“RNA-seq”; 36; S = 0.886; 2018) (Jansen et al., 2019) in 2022. “lncRNA” became cluster #4 (“NEAT1”; 38; S = 0.857; 2018) (Riva et al., 2016). “Epigenetics” became cluster #8 (“microglia”; 44; S = 0.921; 2018) (Jansen et al., 2019), cluster #10 (‘epigenetic clock’; 22; S = 0.999; 2014) (Levine et al., 2018), cluster #12 (“deoxyribonucleic acid methylation”; 21; S = 0.997; 2014) (Ritchie et al., 2015), and cluster #2 (“exosomes”; 40; S = 0.881; 2018) (Kalluri and LeBleu, 2020) in 2022.

#### Most cited papers and turning-point papers

We present the top 10 most co-cited references in Table 1. The review by Bartel DP in Cell is the most co-cited, with 132 citations in our network and 15077 citations in the literature, elaborating on the impact of miRNAs on both gene expression and evolution (Bartel, 2009). The second-most cited paper is a meta-analysis on genome-wide association studies of Alzheimer’s disease published by Lamberty et al. (Lambert et al., 2013), with 102 citations in our network, compared with 2558 citations in the literature. Additionally, the most co-cited paper in our network is an epigenomic study of AD published by De Jager et al. in 2014 in Nature Neuroscience (De Jager et al., 2014), with 175 citations in our network and 537 citations in the literature. The case-control study of changes in microRNA expression profiles of sporadic AD patients is the second-most cited article published by Hebert et al. in the National Academy of Sciences of the USA, with 141 citations in our network and 860 citations in the literature (Hébert et al., 2008). Furthermore, we extracted the top 25 most co-cited references from the last 5 years, shown in Supplementary Table 2U. De Jager et al.’s epigenomic study10 is the most cited paper, followed by the cohort study on neuropathy-associated regulation of mRNA targets published by Lau et al. (2013), and the DNA methylation age predictor proposed by Horvath in 2013 (Horvath, 2013).

**TABLE 1:** The top 10 most cited references.

Number of citations in the network	Number of citations in the literature	Cited reference	Year	Source	Vol	Page	Title	Doi	Type of paper	Related cluster in Figure 1
175	537	De Jager PL	2014	NAT NEUROSCI	17	1156	Alzheimer’s disease: early alterations in brain DNA methylation at ANK1, BIN1, RHBDF2 and other loci	10.1038/nn.3786	The Basic Research	2
141	860	Hebert SS	2008	P NATL ACAD SCI USA	105	6415	Loss of microRNA cluster miR-29a/b-1 in sporadic Alzheimer’s disease correlates with increased BACE1/beta-secretase expression	10.1073/pnas.0710263105	The Basic Research	1
132	15,077	Bartel DP	2009	CELL	136	215	MicroRNAs: Target Recognition and Regulatory Functions	10.1016/j.cell.2009.01.002	Review	1
122	322	Lunnon K	2014	NAT NEUROSCI	17	1164	Methylomic profiling implicates cortical deregulation of ANK1 in Alzheimer’s disease	10.1038/nn.3782	The Basic Research	2
122	605	Wang WX	2008	J NEUROSCI	28	1213	The expression of microRNA miR-107 decreases early in Alzheimer’s disease and may accelerate disease progression through regulation of beta-site amyloid precursor protein-cleaving enzyme 1	10.1523/JNEUROSCI.5065-07.2008	The Basic Research	1
122	550	Graff J	2012	NATURE	483	222	An epigenetic blockade of cognitive functions in the neurodegenerating brain	10.1038/nature10849	The Basic Research	4
119	1162	Guan JS	2009	NATURE	459	55	HDAC2 negatively regulates memory formation and synaptic plasticity	10.1038/nature07925	The Basic Research	4
108	677	Peleg S	2010	SCIENCE	328	753	Altered Histone Acetylation Is Associated with Age-Dependent Memory Impairment in Mice	10.1126/science.1186088	The Basic Research	4
104	322	Lau P	2013	EMBO MOL MED	5	1613	Alteration of the microRNA network during the progression of Alzheimer’s disease	10.1002/emmm.201201974	The Basic Research	0
102	2,558	Lambert JC	2013	NAT GENET	45	1452	Meta-analysis of 74,046 individuals identifies 11 new susceptibility loci for Alzheimer’s disease	10.1038/ng.2802	Meta-analysis	2

Additionally, we analyzed the top 3 references with the strongest beginning of citation bursts (Hébert et al., 2008; Wang et al., 2008; Kim et al., 2007) (Supplementary Tables 2S, T), revealing that MicroRNAs play an important function in post-transcriptional regulation of gene expression and contribute to the progression of NDs (e.g., Alzheimer’s disease, Parkinson’s disease). When focusing on the last 5 years, the corresponding citations were the prospective cohort study of DNA methylation changes in relation to neuropathology by De Jager et al. in 2014 (De Jager et al., 2014), the cohort study on miRNAs altered in AD by Lau et al. (2013), and the introduction of DNA methylation age by Horvath (2013), thus proving the important role of novel epigenetic control (Supplementary Tables 2U, V).

Importantly, we identified intellectual “turning-point” papers associated with significant contributions to domain advancement, such as Harper et al. research, central to cluster #5 (“RNA interference”) (Harper et al., 2005), which revealed RNA interference’s general applicability to treating other dominant neurodegenerative disorders; the papers by Guan (Guan et al., 2009) and Graff (Gräff et al., 2012) in #4 (“RNA methylation”), essential to epigenetic dysfunction of genes in vulnerable neurons, further developing cluster #2 (“RNA methylation”) and cluster #6 (“epigenetics”) with updated evidence showing methylomic variation associated with neurodegenerative disorders. Recently, various studies related to NDs, such as MS (Junker et al., 2009), AD (Mastroeni et al., 2010), ALS (DeJesus-Hernandez et al., 2011), and PD (Poewe et al., 2017), have demonstrated the important role of epigenetic regulation in molecular genetic mechanisms.

#### Co-occurring author keywords networks

**FIGURE 2:**
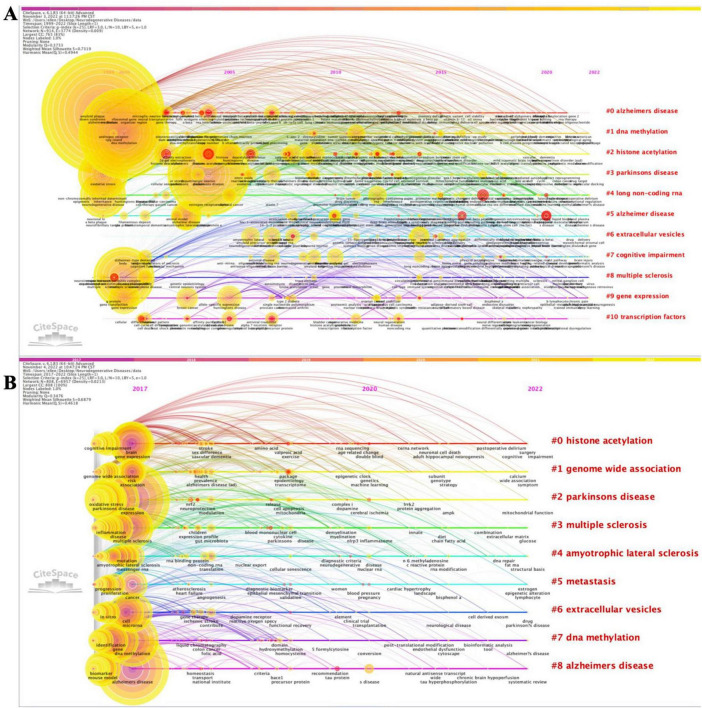
Timeline visualization of co-occurring author keywords networks [**(A)** 1999–2022 and **(B)** 2017–2022]. The nodes represent keywords, and the colors show the average year of publication for each node. The size of tree ring is proportional to the burstness of keyword co-occurrence. The co-occurrence network is weighted on total link strength across different keyword nodes and scored on the average publication years. The clusters are labeled in red at the far right of the timeline maps.

Analyzing the most cited keywords, we examined research hotspots and trends. We constructed the timeline of the co-occurring authors’ keyword network (1999–2022) using CiteSpace (Figure 2A). Nine clusters of keywords were identified, with the most important being “Alzheimer’s disease”, followed by “DNA methylation”, “histone acetylation”, “Parkinson’s disease”, “long non-coding RNA”, “extracellular vesicles”, “cognitive impairment”, “multiple sclerosis”, “gene expression”, and “transcription factors”. Furthermore, we extracted the same network while focusing on the 2017–2022 period (Figure 2B), identifying eight clusters. The most important cluster was “histone acetylation”, followed by “genome-wide association”, “Parkinson’s disease”, “multiple sclerosis”, “amyotrophic lateral sclerosis”, “metastasis”, “extracellular vesicles”, “DNA methylation”, and “Alzheimer’s disease”. Both co-occurring author keywords networks (1999–2022 and 2017–2022) presented a significant silhouette score (S > 0.7) and an acceptable modularity score (Q > 0.3). Moreover, the results for burstness revealed that the four most cited keywords ranked by the beginning of citation bursts were “gene therapy” (1999), “RNA interference” (1999), “gene silencing” (1999), and “histone deacetylase inhibitor” (1999) (Supplementary Tables 2W, X). When considering the last 5 years (in the 2017–2022 time period), our data revealed that the last keywords with the latest beginning of citation bursts were “personalized medicine”, “peripheral blood”, “protein-protein interaction”, “promoter methylation”, “machine learning”, and “valproic acid” (Supplementary Tables 2Y, Z).

We further extracted the overlay of visualization for the co-occurring author keywords networks based on the average publication years (1999–2022 time period) with VOSviewer (Supplementary Figure 7). The most cited keywords were “Alzheimer’s disease”, “epigenetics”, “Parkinson’s disease”, “DNA methylation”, and “microRNA”. Some keywords reflecting the latest trends of research were ‘biomarkers’, ‘exosomes’, ‘environment’, and ‘neurons’.

### Publication outputs and major journals

Our dataset initially comprised 16,181 references. Following the detailed data filtering process outlined in our protocol, 1,075 references were excluded. The final dataset included 15,661 studies (12,314 articles and 3,031 reviews) in 16 different languages, spanning from 1999 to 2022. On average, there were 1.47 authors per publication across 635 different journals (Supplementary Table 6). The earliest article identified in our analysis was a clinical case study by Nasreddine et al. in 1999, focusing on mutational analysis of the tau coding region in frontotemporal dementia, suggesting a potential role of genetic or epigenetic factors (Nasreddine et al., 1999).

As depicted in Supplementary Figure 8, we investigated the annual scientific production and average citations per year for the references. Our analysis revealed a steady increase in annual scientific production since 1999, particularly accelerating from 2003 onwards. The average annual growth rate was 85.7%, with publications per year rising from 52 in 2003 to a peak of 1,785 articles in 2021 and maintaining a high level of 1,393 articles per year until 2022. Moreover, the average citations per document per year showed an upward trend over the same period, reaching a peak of 11.52 in 2002 and 5.51 in 2021.

Regarding journals, we identified the top five journals with the most references during the 1999–2022 period (Supplementary Figure 9A), namely PLOS One (*n* = 3,027), Journal of Biological Chemistry (*n* = 2,046), Journal of Alzheimer’s Disease (*n* = 1,383), Molecular Neurobiology (*n* = 1,055), and International Journal of Molecular Sciences (*n* = 1,185). Over the past 5 years, the most referenced journals were International Journal of Scientific Reports (*n* = 805), Scientific Reports (*n* = 720), Molecular Neurobiology (*n* = 602), Journal of Alzheimer’s Disease (*n* = 430), and Frontiers in Neuroscience (*n* = 365) (Supplementary Figure 9B). Furthermore, we extracted the co-cited journal network and identified the five journals with the highest number of publications over the past 30 years, namely Proceedings of the National Academy of Sciences of the United States of America, Nature, Journal of Biological Chemistry, PLOS One, and Cell, among others (Supplementary Figure 10).

### Analysis of cooperation networks across countries and institutions

**FIGURE 3:**
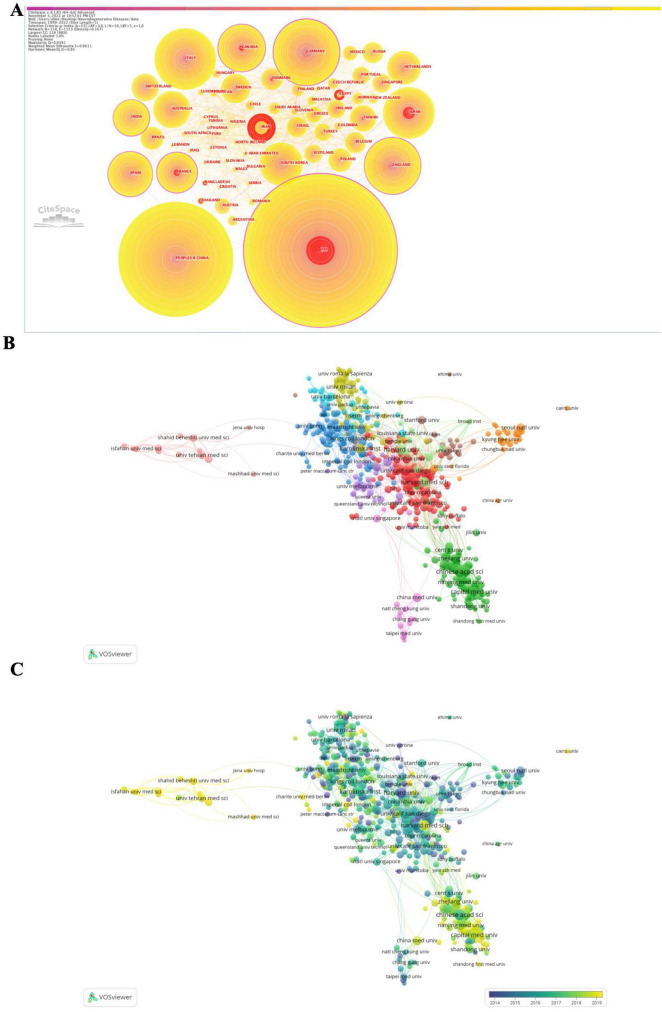
Network of the co-authors’ countries [1999–2022, **(A)**], and the network of co-authors institutions [2017–2022, **(B)**] with scored on the average publication year [2017–2022, **(C)**] for epigenetics on neurodegenerative diseases obtained with CiteSpace and VOSviewer. Minimum number of documents of an institution = 15, 512 meet the thresholds, which are represented within 11 clusters.

The most cited countries and institutions are summarized in Supplementary Table 5 and Figures 3A–C. We identified 111 countries, with Germany exhibiting the highest degree of centrality, followed by the United States of America (USA), India, France, and Canada. In terms of total citations by country, the USA contributed the most to global research of epigenetics on neurodegenerative diseases (ENDs) with 5,016 citations, followed by the Republic of China, Germany, Italy, and England.

A total of 513 different organizations were identified, with the top five institutions by citation counts being the Chinese Academy of Sciences (226 citations), Harvard School (186), Shanghai Jiao Tong University (179), Fudan University (170), and Harvard Medical School (168). The top five institutions/affiliations with the highest centrality were Harvard School, followed by Johns Hopkins University, University of Pennsylvania, Emory University, and Boston University.

Furthermore, when restricting the analysis to the last 5 years (2017–2022), we found that Germany still exhibited the highest degree of centrality. The top five most cited countries were the Republic of China (2,944 citations), USA, Italy, Germany, and England. The top five most cited institutions were Harvard Medical School (153 citations), Shanghai Jiao Tong University, Capital Medical University, Chinese Academy of Sciences, and Fudan University.

The analysis of burstness demonstrated that the USA had the strongest citation burst strength throughout all times (79.05). Additionally, the burst detection analysis revealed that Harvard School had the strongest (55.43) and longest citation burst (2002–2017). Some Chinese institutions/affiliations exhibited stronger citation burst strength in the last 5 years, including Central South University, Peking Union Medical College, Zhejiang University, and Nanjing University.

### Analysis of co-authorship network

**FIGURE 4:**
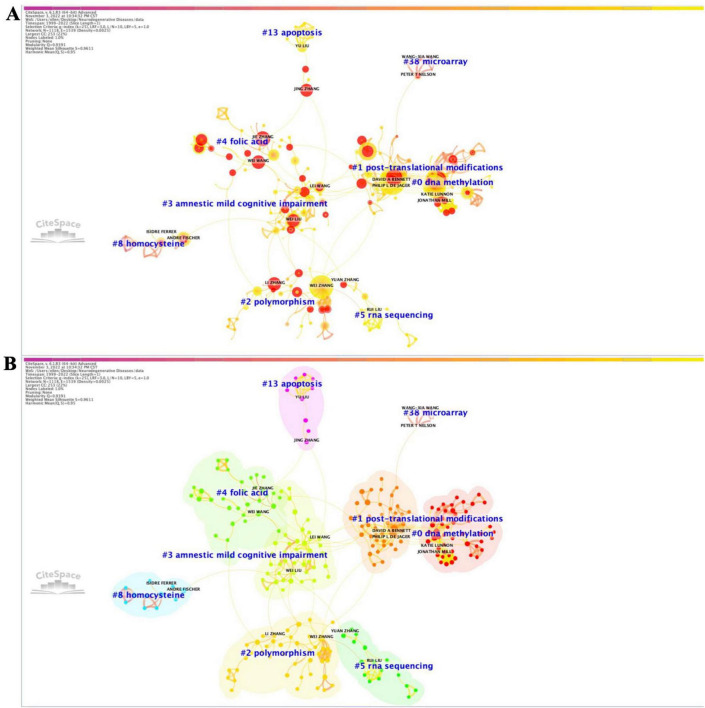
Co-authorship network **(A)** with corresponding clusters **(B)** from 1999 to 2022.

We analyzed authors who published the greatest number of papers associated with ENDs and their collaborative network (Figure 4 and Supplementary Table 3). Co-authorship networks allow for the visualization of scientific collaboration between authors based on the frequency of co-authorship. The co-authorship network showed significant modularity, with a silhouette score indicating highly credible clusters (M = 0.9391; S = 0.9611) (Supplementary Table 4 and Supplementary Figure 12). This network revealed that the most important recent cluster is cluster #0, labeled ‘DNA methylation’, which primarily pertains to the most recent developments in methylomic variation associated with neurodegenerative disorders. De Jager PL and Braak H were identified as two key authors linking cluster #0. The top five co-authors with the strongest citation burst were Mohammad Taheri, Soudeh GF, Wei Wang, Lei Zhang, and Wei Liu; in the last 5 years, the top five authors were Mohammad T, Soudeh GF, Wei Wang, Ying Liu, and Nan Zhang.

In addition, we conducted the co-authorship network using VOSviewer, representing a similar network (Supplementary Figure 11). We further explored citations using the author co-citation network to determine ‘who cites who?’ from 2017 to 2022 in our database.

### Comprehensive analysis of based on bibliometric coupling

**FIGURE 5:**
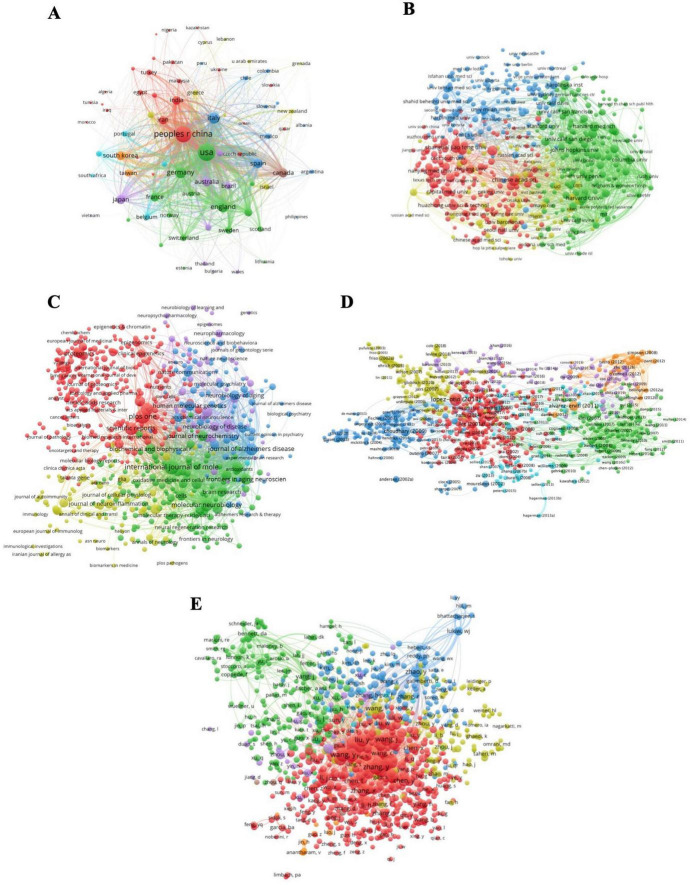
Map of bibliographic coupling analysis based on countries **(A)**, institutions **(B)**, journals **(C)**, references **(D)**, and authors **(E)** (weights on the total link strength). Minimum number of documents of a country = 5, 77 meet the thresholds; Minimum number of citations of a document = 100, 1000 meet the thresholds; Minimum number of documents of a journal = 5, 635 meet the threshold; Minimum number of documents of an author = 10, 934 meet the threshold; Minimum number of documents of an institution = 15, 512 meet the thresholds.

We analyzed bibliometric coupling networks across countries (A), institutions (B), journals (C), references (D), and authors (E) (Figure 5). When two references refer to the same piece of paper, the relationship between them is bibliometric coupling, which is utilized in literature relation research, literature retrieval, and literature structure research. In terms of countries, China and the United States have significant influences in the field of ENDs. Major clusters have formed in institutions, represented by the Chinese Academy of Sciences, Harvard School, Harvard Medical School, Johns Hopkins University, Karolinska Institution, and Shanghai Jiao Tong University. The influence of Chinese and American institutions is significantly higher than that of other countries. We identified influential journals such as PLOS One, International Journal of Molecular Sciences, Scientific Reports, and Journal of Alzheimer’s Disease. In the reference clustering network, influential references include studies by Choudhary et al. (2009), Alvarez-Erviti et al. (2011b), and López-Otín et al. (2013) on epigenetics, published in 2009, 2011, and 2013, respectively. Regarding authors, most influential authors are Chinese (e.g., Yuan Zhang, Ying Liu, and Yan Wang), who exhibit a high degree of cooperation and are core figures in each cooperation network.

## Discussion

### Summary of the main findings in this study

In this study, we conducted a bibliometric analysis of literature on ENDs spanning from 1999 to 2022. We synthesized current trends and hot topics in ENDs research by analyzing leading keywords, countries, institutions, journals, and authors, providing valuable insights for future research in this promising field. Our findings reveal a consistent increase in thematic research from 2002 to 2017, with the annual publication count rising from 28 to 1100 articles. Notably, in 2021–2022, the yearly publication count peaked at 1785 articles, indicating a growing and sustained interest among scholars in ENDs. The United States emerged as the leading contributor in terms of both total publications and citation count over the three decades under investigation. Harvard Medical School, the oldest medical institution in the USA, stood out as the most influential research institute in the field of ENDs. Analyzing journal contributions, we found that the International Journal of Molecular Sciences had the highest number of publications, while Cold Spring Harbor Perspectives in Medicine emerged as the most cited journal in the last 5 years. This underscores the significance of research from the USA and its affiliated publications in shaping the discourse on ENDs. Additionally, there was a notable increase in Science Citation Index publications from Chinese institutions, indicating a growing presence in the field. Examining authorship, we identified the top five most productive and cited authors, including representatives from both developed and developing countries. Furthermore, analysis of collaboration networks revealed an increasing representation of Chinese authors in recent years.

### Trends and future directions in ENDs research

The examination of trends and the future trajectory of ENDs through keyword co-occurrence and reference networks spanning from 1999 to 2022 reveals significant insights into the evolution of research in this field. The analysis elucidates connections among 25 distinct clusters, providing a nuanced understanding of the research landscape regarding posttranscriptional gene control and selective gene transcription expression. Firstly, the co-citation reference networks indicate a consistent upward trend in research on ENDs from 1999 to 2022, offering deeper insights into the control of posttranscriptional gene expression. Notably, recent years have seen heightened attention towards keywords such as ‘dna methylation’, ‘heavy metals’, ‘exosomes’, and ‘microglia’, reflecting emerging research trends and current hotspots of investigation. Furthermore, a global increase in evidence synthesis regarding the underlying pathological mechanisms of ‘dna methylation’, ‘heavy metals’, ‘exosomes’, and ‘microglia’ in relation to epigenetic functions and regulatory mechanisms of Neurological Diseases (NDs) has been identified. This synthesis underscores the significance of understanding processes such as neuroinflammation (Rodríguez-Gómez et al., 2020), mitochondrial dysfunction and energy failure (Song et al., 2022), and clearance mechanisms (Urbizu and Beyer, 2020) in the context of ENDs research.

Moreover, analysis of co-occurring cited keywords and key references reveals a rapid escalation in publications within the field (Song et al., 2022; Hu et al., 2022; Ramírez et al., 2022). The 2022 cited keywords suggest a mediating role of epigenetics in the interactions between the epigenome and environmental elements. This highlights the increasing recognition of epigenetics as a pivotal nexus between gene expression and environmental influences, including pesticides, heavy metals, drugs, and dietary habits (Coppedè et al., 2022; Honkova et al., 2022; Zhang et al., 2022). However, despite advancements in understanding the interaction between hereditary and environmental factors in ENDs, there remains a fundamental knowledge gap regarding the precise mechanisms underlying these processes. Future research endeavors should prioritize addressing this gap to advance the field and pave the way for improved pathological assessment and potential treatment avenues for neurological diseases.

Notwithstanding the remarkable advancements in the realm of epigenetics of ENDs, further research is warranted to bridge two knowledge gaps: (a) plenty of epigenetic effectors with known detrimental effects on the nervous system have been identified to date, whereas available data on cell-type-specific or region-specific epigenetic alternations is relatively sparse. Luckily, the rapid development of single-cell and spatial sequencing techniques allows for a comprehensive mapping of epigenetic modifications of specific genes in an individual cell or an anatomical region of interest. In this case, applying these methods to gain a more in-depth understanding of the epigenetic origin of ENDs and conducting more gain- and loss-of-function experiments using some state-of-art epigenetic editing technologies (e.g., Cre/loxP, CRISPR/Cas9) to realize cell-type-specific epigenetic modulation of hub genes implicated in END development is urgently needed; (b) as demonstrated by our findings, basic research and review articles account for the vast majority of the most cited references (Table 1). By contrast, influential clinical trials and practice guidelines are quite lacking, which seemingly reflects that the wide application of epigenetically targeted therapies from bench to bedside for treating ENDs has not yet been fully achieved at present. Indeed, genetic or drug-induced rodent models of ENDs are powerful tools for validating the therapeutic efficacy and safety of pharmacologically intervening epigenetic effectors, animal models, however, cannot recapitulate the pathophysiologic complexity of END in human patients, which prompt us to shift attention to human-based models or perform more clinical trials to overcome the translational barriers.

### Strengths and limitations of bibliometric analysis

This study employs three different software packages for bibliometric analysis, offering a comprehensive overview of ENDs research over the past decade. Bibliometric analysis serves as a valuable tool for evaluating the breadth of publications within a research discipline and identifying patterns and trends in research output. As demonstrated in our results, scientometric analysis provides insights into the knowledge structure, topic evolution, and research hotspots of ENDs. However, it is crucial to acknowledge the inherent limitations of such analyses. These include selection bias, as the datasets primarily consist of English-language publications, potentially excluding research from non-English-speaking countries. Additionally, publication bias may skew results towards studies with significant findings, while time constraints may lead to underestimation of the value of newly published articles.

Furthermore, the focus on primary information such as the number of publications and first authors may not fully capture the influence of references or authors in the field. Despite these limitations, bibliometric analysis remains a valuable tool for understanding the landscape of ENDs research and identifying areas for future investigation.

## Conclusion

Collectively, this is the first scientometric study on ENDs, and these findings may mark a new step on the path toward the field. Compared to reviews and meta-analysis, scientometric analyses will be valuable in future studies to discover epigenetics associated with NDs, comprehensively guiding clinicians and scholars on the history of research and emerging trends. Also, as shown in our study, the scientometric analyses can provide a synopsis of questions in relation to clinical and scientific research, potentially informing future trials. Moreover, this work can help scholars to identify the most representative and authoritative authors and journals in the field of ENDs. The high number of recent publications is driven by corresponding state policies and fund input. In terms of cooperation, domestic partnerships are arising, with underrepresented international cooperation. We believe that in the future, more researchers will continue to probe this problem and achieve new development in the field of ENDs.

## Supplementary material

Supplementary Figure 1 Flow chart of the scientometric study.

Supplementary Figure 2 Detail focus on most important clusters of the co-citation reference networks ranked by burstness of citations (1999–2022). For each cluster, we report all five top keywords obtained, the selected label being the keywords that are the most cited (generated by the likelihood ratio of keywords). These keywords are highly susceptible to represent the overall topic of a cluster. Burstness is represented in each cluster with red tree-rings around nodes.

Supplementary Figure 3 Link walkthrough between clusters based on burstness dynamic for co-cited reference network (1999–2022).

Supplementary Figure 4 Network of co-cited reference **(A)** with corresponding clusters **(B)** and timeline view (C) for the 20172022 time period.

Supplementary Figure 5 Detail focus on all 14 extracted clusters of the co-citation reference networks ranked by burstness of citations for the time period 2017–2022.

Supplementary Figure 6 Network of co-cited reference **(A)** with corresponding clusters **(B)** and timeline view (C) for the year 2022.

Supplementary Figure 7 Overlay visualization of co-occurring authors’ keywords **(A)**, and scored on the average publication year **(B)**. Minimum number of occurrences of a keyword = 10, 837 meet the thresholds, which are represented within 10 clusters. The nodes represent keywords and the colors show the average year of publication for each node. The size of a node is proportional to the frequency of keyword co-occurrence. The co-occurrence network is weighted on total link strength across different keyword node, and scored on the average publication years from 1999 to 2022.

Supplementary Figure 8 Annual scientific production **(A)** and average citation per year for references **(B)** (1999–2022).

Supplementary Figure 9 Top 10 growth source (1999–2022 and 2017–2022).

Supplementary Figure 10 Overlay visualization of most cited journals for the last 5 years **(A)**, and most co-cited journals which published the most articles these last 30 years **(B)**. Figure A is obtained with CiteSpace and Figure B with VOSviewer. **(A)** 13 clusters are identified. **(B)** Weighted on documents, Minimum number of citations of a journal = 150, 892 meet the thresholds, which are identified with 7 clusters.

Supplementary Figure 11 Co-authorship network obtained with VOSviewer. 25 clusters are identified comprising 489 different authors. Each cluster is identified with a different color. Minimum number of documents of an author = 8.

Supplementary Figure 12 Visualization of the author co-citation network **(A)**, with corresponding clusters **(B)** and time map **(C)** (2017–2022).

Supplementary Table 1 Co-cited reference network detailed clusters (1999–2022, 2017–2022 and 2022).

Supplementary Table 2 Burstness analysis for countries, institutions, authors, references and keywords (1999–2022, 2017–2022). The beginning of a blue line depicts when an article is published. The beginning of a red segment marks the beginning of a period of burst, whereas the end of the red segment marks the end of the burst period.

Supplementary Table 3 Summary of the largest clusters identified for co-authorship network (1999–2022) obtained with CiteSpace.

Supplementary Table 4 Summary of the largest clusters identified for the author co-citation network (2017–2022) obtained with CiteSpace.

Supplementary Table 5 The top countries and institutions (1999–2022 and 2017–2022 period).

Supplementary Table 6 Average citations and total link strength of countries, institutions, journals, references, and authors per cluster based on bibliographic coupling analysis.
