# Supplementary material for: Scoping review of epigenetics on neurodegenerative diseases: research frontiers and publication status
Source: Front Neurosci. 2024 Oct 9;18:1414603. doi: 10.3389/fnins.2024.1414603 (PMC11496254; doi:10.3389/fnins.2024.1414603)
Supplement: Supplementary file 1 [file Data_Sheet_7.DOCX]

The researchers will perform a wide-ranging search for related publications that consists of all field terms and phrases relevant to epigenetics on neurodegenerative diseases. Following a careful selection and validation of search terms in WOSCC, we plan to search WOSCC from inception using the combination of keywords and MeSH terms: TS=(Epigenomics OR Epigenomic OR Epigenetics OR Epigenetic OR “DNA Methylation” OR “DNA Methylations” OR “Methylation, DNA” OR “Methylations, DNA” OR “Histone Acetylation” OR “Histone Methylation” OR “Histone Modification” OR “Histone Ubiquitination” OR “Histone Ubiquitylation” OR “Histone Sumoylation” OR “Histone Sumoylations” OR “Histone Sumo Conjugation” OR “Histone Sumo-Conjugations” OR “Histone ADP-Ribosylation” OR “Histone Variants” OR “Histone H2a.F” OR “RNA Methylation” OR “RNA modification” OR “Chromatin Assembly” OR “Chromatin Assembly and Disassembly” OR “Chromatin Remodeling” OR Epitranscriptomic OR Epitranscriptomics OR “RNA, Untranslated” OR “Untranslated RNA” OR npcRNA OR “RNA, Nontranslated” OR “Nontranslated RNA” OR “RNA, Non-Peptide-Coding” OR “Non-Peptide-Coding RNA” OR “RNA, Non Peptide Coding” OR “RNA, Non-Protein-Coding” OR “Non-Protein-Coding RNA” OR “RNA, Non Protein Coding” OR “RNA, Noncoding” OR “Noncoding RNA” OR “RNA, Non-Coding” OR “Non-Coding RNA” OR “RNA, Non Coding” OR “RNA, Small Nuclear” OR snRNA OR “Small Nuclear RNA” OR “Low Molecular Weight Nuclear RNA” OR “Small Molecular Weight RNA” OR “Chromatin-Associated RNA” OR “Chromatin Associated RNA” OR “RNA, Chromatin-Associated” OR “RNA, Small Nucleolar” OR” Small Nucleolar RNA” OR snoRNA OR “telomerase RNA” OR TERC OR “TERC RNA, human” OR “telomerase RNA component, human” OR bTR OR “bovine telomerase RNA” OR “telomerase RNA component” OR “TERC RNA” OR “hTR RNA” OR MicroRNAs OR MicroRNA OR miRNAs OR miRNA OR “Primary MicroRNA” OR “MicroRNA, Primary” OR “Primary miRNA” OR “miRNA, Primary” OR pri-miRNA OR “RNA, Small Temporal” OR “Temporal RNA, Small” OR stRNA OR “Small Temporal RNA” OR pre-miRNA OR “RNA, Small Interfering” OR “Interfering RNA, Small” OR “Short Interfering RNA” OR “Interfering RNA, Short” OR “RNA, Short Interfering” OR “Small Interfering RNA” OR siRNA OR “Piwi-Interacting RNA” OR “Piwi Interacting RNA” OR “RNA, Piwi-Interacting” OR “RNA, Piwi Interacting” OR piRNA OR “Short Hairpin RNA” OR “Hairpin RNA, Short” OR “RNA, Short Hairpin” OR “Small Hairpin RNA” OR “Hairpin RNA, Small” OR “RNA, Small Hairpin” OR shRNA OR “Repeat-Associated siRNA” OR “Repeat Associated siRNA” OR “siRNA, Repeat-Associated” OR “siRNA, Repeat Associated” OR “Scan RNA” OR scnRNA OR “Small Scan RNA” OR “RNA, Small Scan” OR “Scan RNA, Small” OR “RNA, Scan” OR “Trans-Acting siRNA” OR “Trans Acting siRNA” OR tasiRNA OR “siRNA, Trans-Acting” OR “siRNA, Trans Acting” OR “RNA, Long Noncoding” OR “Noncoding RNA, Long” OR lncRNA OR “Long ncRNA” OR “ncRNA, Long” OR “RNA, Long Non-Translated” OR “Long Non-Translated RNA” OR “Non-Translated RNA, Long” OR “RNA, Long Non Translated” OR “Long Non-Coding RNA” OR “Long Non Coding RNA” OR “Non-Coding RNA, Long” OR “RNA, Long Non-Coding” OR “Long Non-Protein-Coding RNA” OR “Long Non Protein Coding RNA” OR “Non-Protein-Coding RNA, Long” OR “RNA, Long Non-Protein-Coding” OR “Long Noncoding RNA” OR “RNA, Long Untranslated” OR “Long Untranslated RNA” OR “Untranslated RNA, Long” OR “Long ncRNAs” OR “ncRNAs, Long” OR “Long Intergenic Non-Protein Coding RNA” OR “Long Intergenic Non Protein” OR “Coding RNA” OR LincRNA OR “LINC RNA” OR LincRNAs OR “RNA, Circular” OR circRNAs OR “Closed Circular RNA” OR “Circular RNA, Closed” OR “RNA, Closed Circular” OR “Circular RNA” OR “Circular RNAs” OR “RNAs, Circular” OR circRNA OR “Circular Intronic RNA” OR “Intronic RNA, Circular” OR “RNA, Circular Intronic” OR ciRNA) AND TS=(“Neurodegenerative Diseases” OR “Neurodegenerative Disease” OR “Degenerative Diseases, Neurologic” OR “Neurologic Degenerative Disease” OR “Degenerative Neurologic Diseases” OR “Degenerative Neurologic Disease” OR “Neurologic Disease, Degenerative” OR “Neurologic Diseases, Degenerative” OR “Nervous System Degenerative Diseases” OR “Neurodegenerative Disorders” OR “Neurodegenerative Disorder” OR “Neurologic Degenerative Conditions” OR “Degenerative Condition, Neurologic” OR “Degenerative Conditions, Neurologic” OR “Neurologic Degenerative Condition” OR “Neurologic Degenerative Diseases” OR “Degenerative Diseases, Nervous System” OR “Degenerative Neurologic Disorders” OR “Degenerative Neurologic Disorder” OR “Neurologic Disorder, Degenerative” OR “Neurologic Disorders, Degenerative” OR “Degenerative Diseases, Spinal Cord” OR “Degenerative Diseases, Central Nervous System” OR “Alzheimer Disease” OR “Alzheimer Syndrome” OR “Alzheimer-Type Dementia” OR “ATD” OR “Alzheimer Type Dementia” OR “Dementia, Alzheimer-Type” OR “Primary Senile Degenerative Dementia” OR “Dementia, Primary Senile Degenerative” OR “Alzheimer Type Senile Dementia” OR “Alzheimer Dementia” OR “Alzheimer Dementias” OR “Dementia, Alzheimer” OR “Alzheimer's Disease” OR “Dementia, Senile” OR “Senile Dementia” OR “Dementia, Alzheimer Type” OR “Alzheimer Type Dementia” OR “Senile Dementia, Alzheimer Type” OR “Alzheimer Sclerosis” OR “Sclerosis, Alzheimer” OR “Alzheimer's Diseases” OR “Alzheimer Diseases” OR “Alzheimers Diseases” OR “Acute Confusional Senile Dementia” OR “Senile Dementia, Acute Confusional” OR “Dementia, Presenile” OR “Presenile Dementia” OR “Alzheimer Disease, Late Onset” OR “Late Onset Alzheimer Disease” OR “Alzheimer's Disease, Focal Onset” OR “Focal Onset Alzheimer's Disease” OR “Familial Alzheimer Disease” OR “FAD” OR “Alzheimer Disease, Familial” OR “Familial Alzheimer Diseases” OR “Alzheimer Disease, Early Onset” OR “Early Onset Alzheimer Disease” OR “Presenile Alzheimer Dementia” OR “Dementia” OR “Dementias” OR “Amentia” OR “Amentias” OR “Senile Paranoid Dementia” OR “Dementias, Senile Paranoid” OR “Paranoid Dementia, Senile” OR “Paranoid Dementias, Senile” OR “Senile Paranoid Dementias” OR “Familial Dementia” OR “Dementia, Familial” OR “Dementias, Familial” OR “Familial Dementias” OR “Parkinson Disease” OR “Idiopathic Parkinson's Disease” OR “Lewy Body Parkinson's Disease” OR “Parkinson's Disease, Idiopathic” OR “Parkinson's Disease, Lewy Body” OR “Parkinson Disease, Idiopathic” OR “Parkinson's Disease” OR “Idiopathic Parkinson Disease” OR “Lewy Body Parkinson Disease” OR “Primary Parkinsonism” OR “Parkinsonism, Primary” OR “Paralysis Agitans” OR “Cognitive Dysfunction” OR “Cognitive Dysfunctions” OR “Dysfunction, Cognitive” OR “Dysfunctions, Cognitive” OR “Cognitive Impairments” OR “Cognitive Impairment” OR “Impairment, Cognitive” OR “Impairments, Cognitive” OR “Mild Cognitive Impairment” OR “Cognitive Impairment, Mild” OR “Cognitive Impairments, Mild” OR “Impairment, Mild Cognitive” OR “Impairments, Mild Cognitive” OR “Mild Cognitive Impairments” OR “Mild Neurocognitive Disorder” OR “Disorder, Mild Neurocognitive” OR “Disorders, Mild Neurocognitive” OR “Mild Neurocognitive Disorders” OR “Neurocognitive Disorder, Mild” OR “Neurocognitive Disorders, Mild” OR “Cognitive Decline” OR “Cognitive Declines” OR “Decline, Cognitive” OR “Declines, Cognitive” OR “Mental Deterioration” OR “Deterioration, Mental” OR “Deteriorations, Mental” OR “Mental Deteriorations” OR “Multiple Sclerosis” OR “Sclerosis, Multiple” OR “Sclerosis, Disseminated” OR “Disseminated Sclerosis” OR MS OR “Multiple Sclerosis, Acute Fulminating” OR “Multiple Sclerosis” OR “Sclerosis, Multiple” OR “Sclerosis, Disseminated” OR “Disseminated Sclerosis” OR MS OR “Multiple Sclerosis, Acute Fulminating”)
