# Supplementary figures and images for: Scoping review of epigenetics on neurodegenerative diseases: research frontiers and publication status
Source: Front Neurosci. 2024 Oct 9;18:1414603. doi: 10.3389/fnins.2024.1414603 (PMC11496254; doi:10.3389/fnins.2024.1414603)

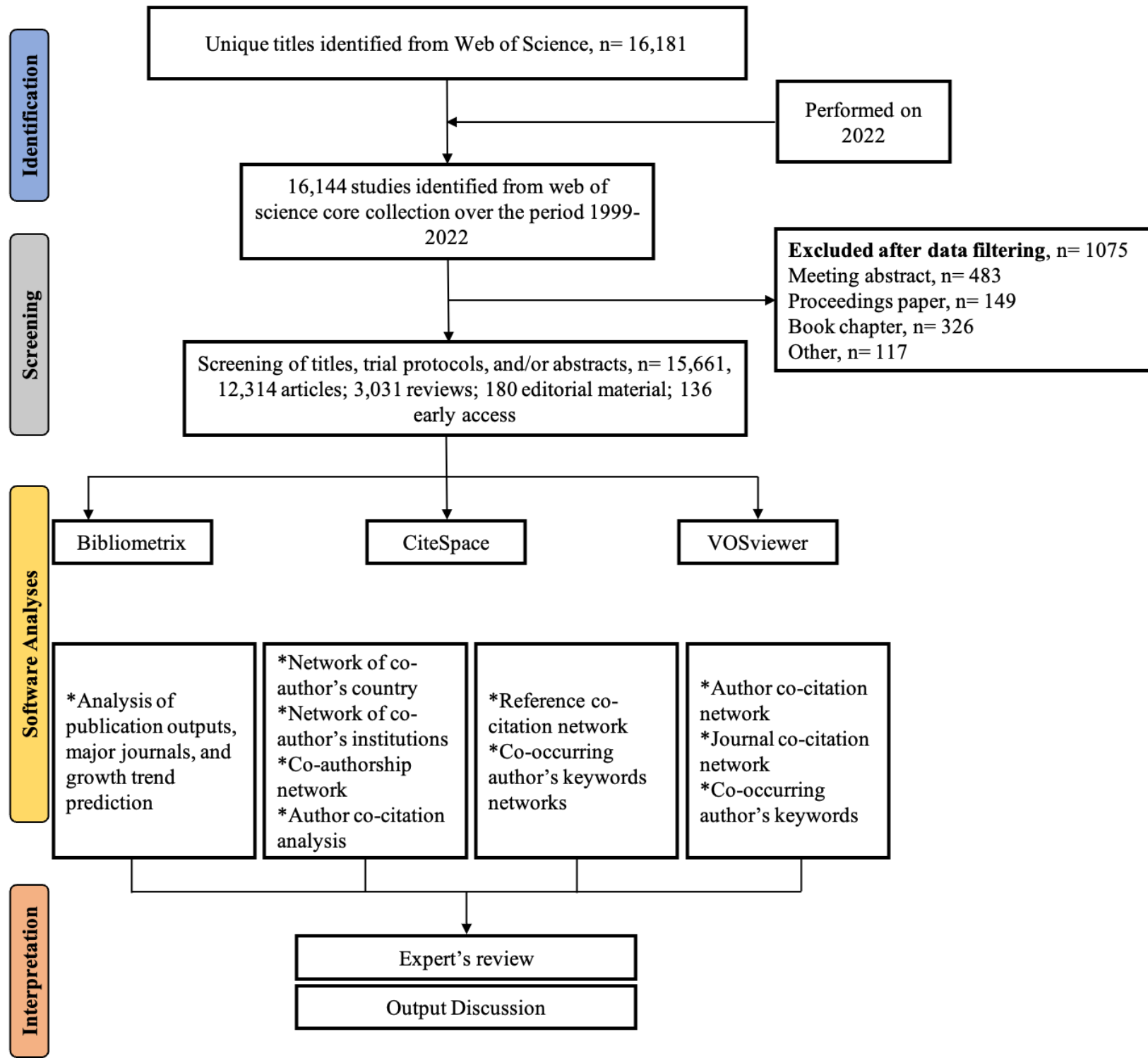

Supplement: Supplementary Figure 1 — Flow chart of the scientometric study. [file Image_1.PDF]

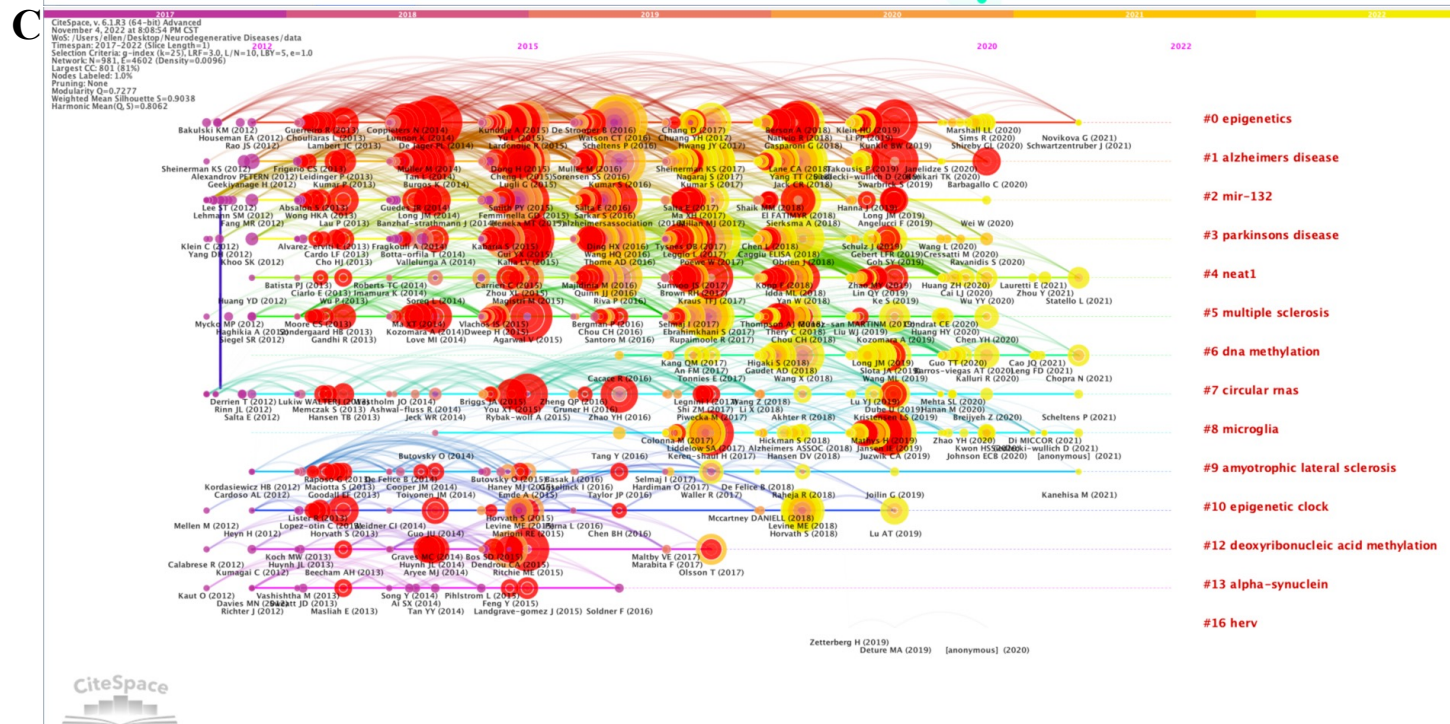

Supplement: Supplementary Figure 4 — Network of co-cited reference (A) with corresponding clusters (B) and timeline view (C) for the 20172022 time period. [file Image_4.PDF]

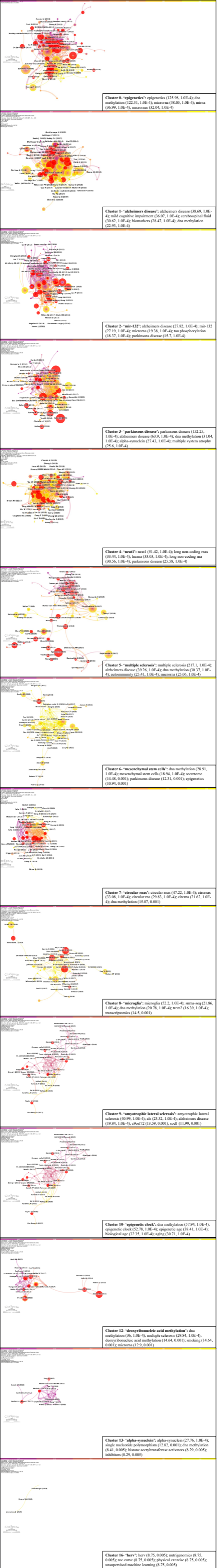

Supplement: Supplementary Figure 5 — Detail focus on all 14 extracted clusters of the co-citation reference networks ranked by burstness of citations for the time period 2017–2022. [file Image_5.PDF]

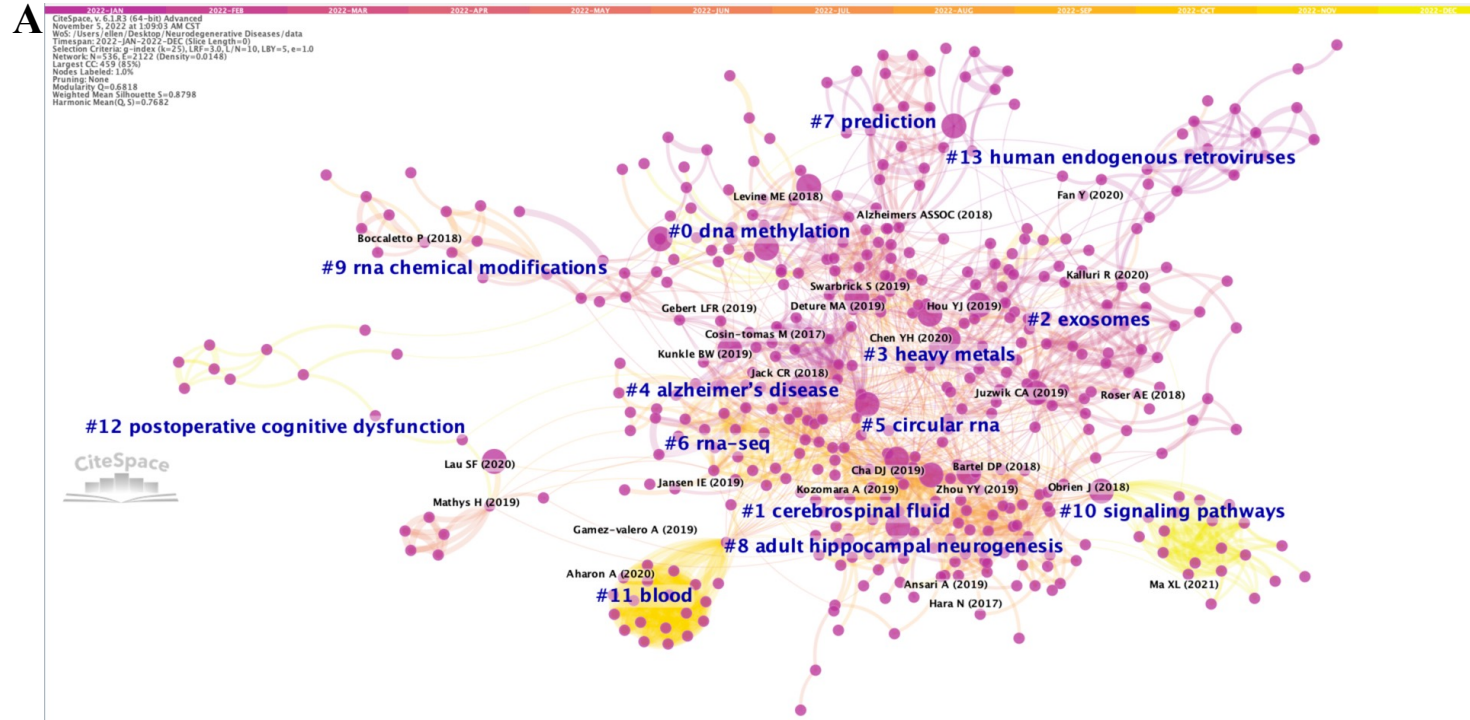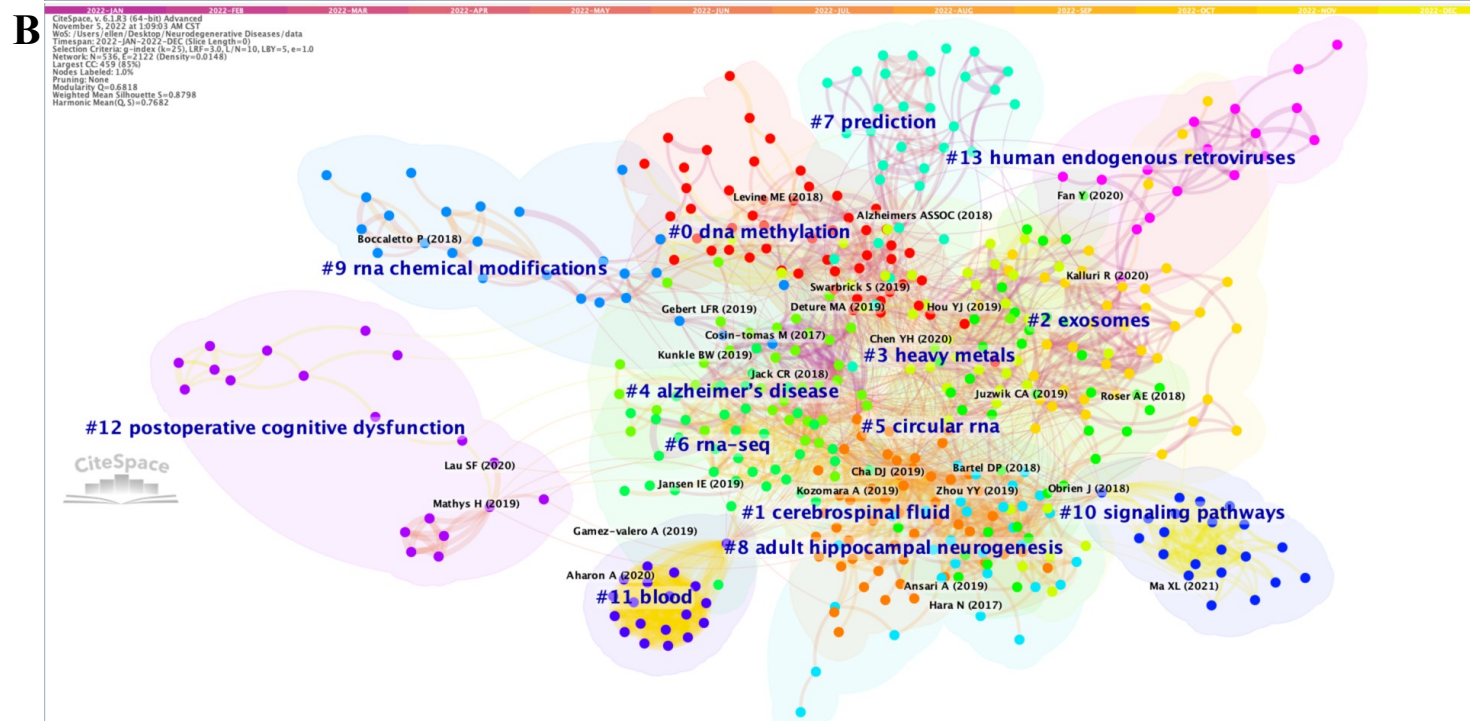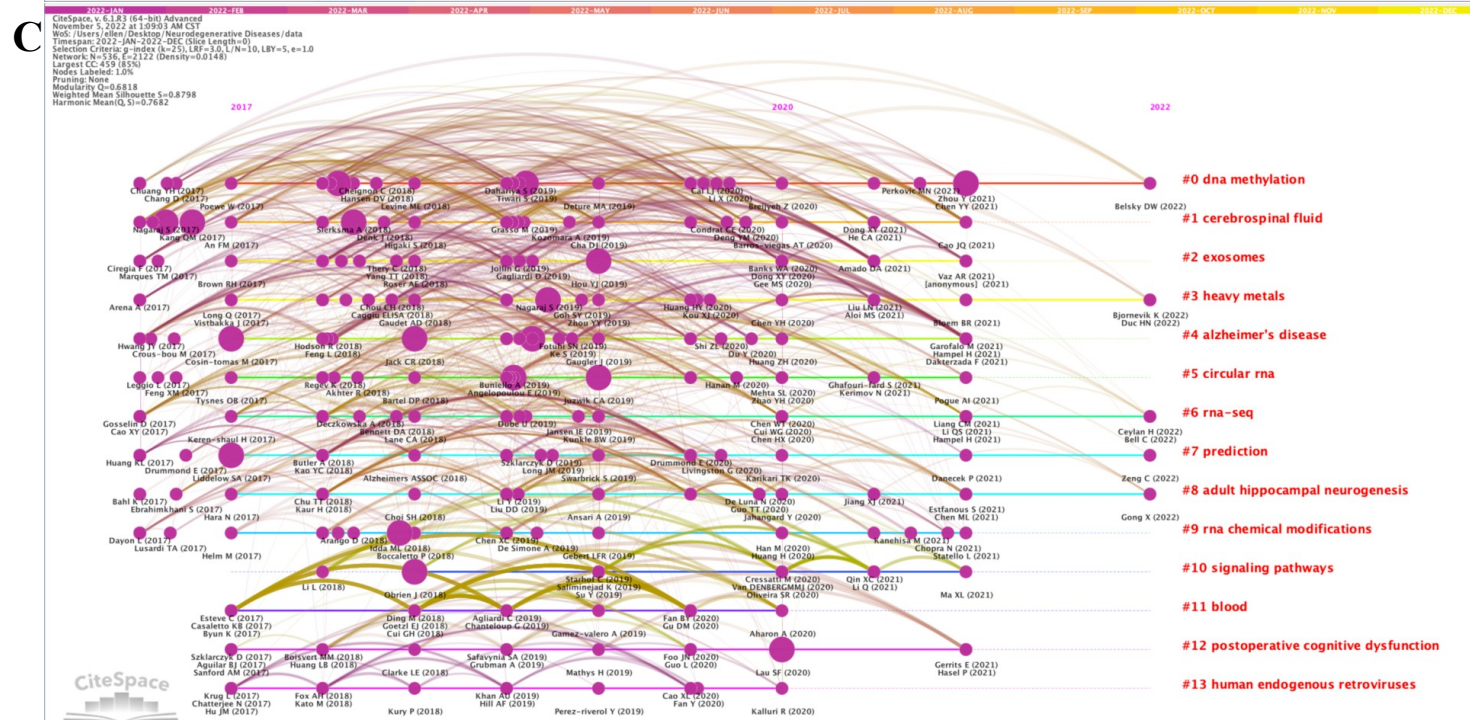

Supplement: Supplementary Figure 6 — Network of co-cited reference (A) with corresponding clusters (B) and timeline view (C) for the year 2022. [file Image_6.PDF]

# B

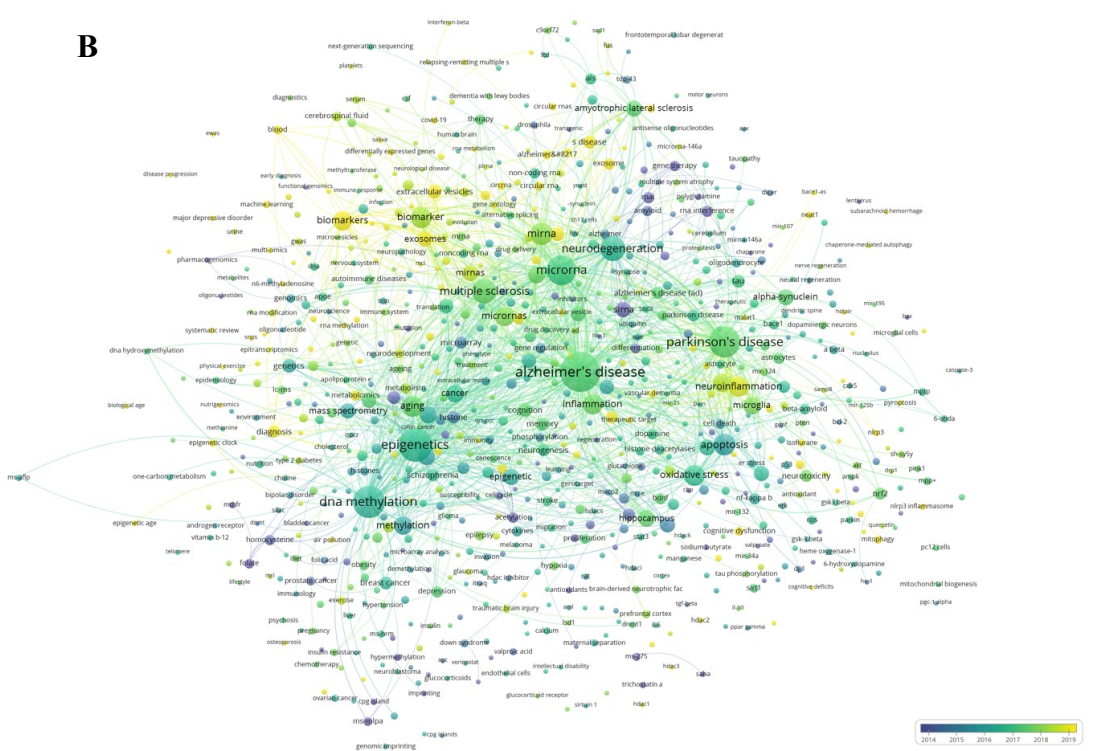

Supplement: Supplementary Figure 7 — Overlay visualization of co-occurring authors’ keywords (A), and scored on the average publication year (B). Minimum number of occurrences of a keyword = 10, 837 meet the thresholds, which are represented within 10 clusters. The nodes represent keywords and the colors show the average year of publication for each node. The size of a node is proportional to the frequency of keyword co-occurrence. The co-occurrence network is weighted on total link strength across different keyword node, and scored on the average publication years from 1999 to 2022. [file Image_7.PDF]

**A**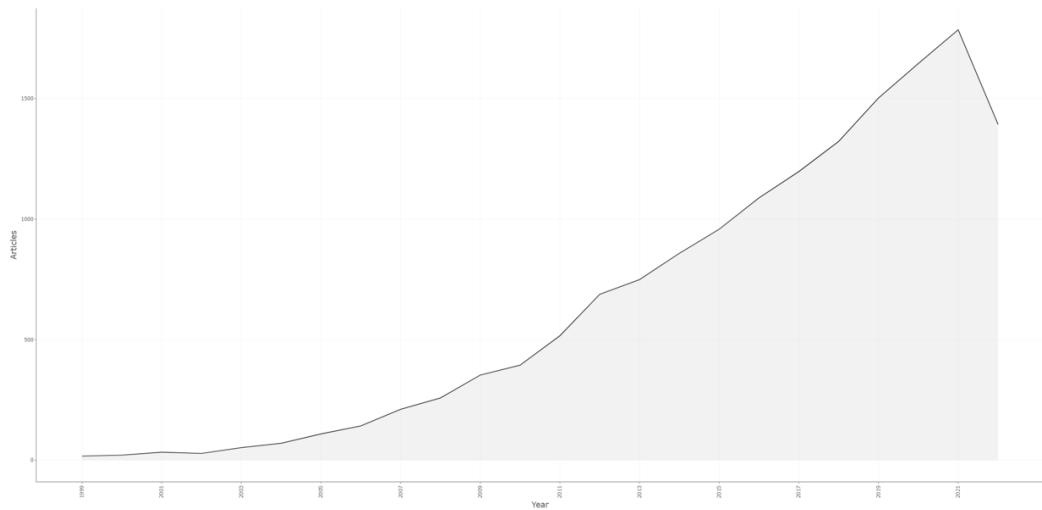**B**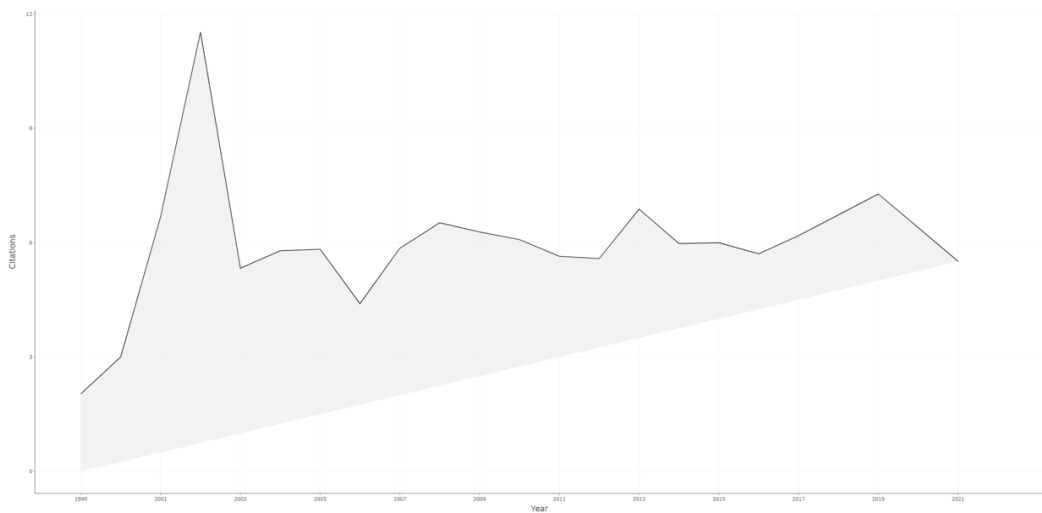

Supplement: Supplementary Figure 8 — Annual scientific production (A) and average citation per year for references (B) (1999–2022). [file Image_8.PDF]
