# Supplementary material for: Scoping review of epigenetics on neurodegenerative diseases: research frontiers and publication status
Source: Front Neurosci. 2024 Oct 9;18:1414603. doi: 10.3389/fnins.2024.1414603 (PMC11496254; doi:10.3389/fnins.2024.1414603)
Supplement: Supplementary Figure 2 — Detail focus on most important clusters of the co-citation reference networks ranked by burstness of citations (1999–2022). For each cluster, we report all five top keywords obtained, the selected label being the keywords that are the most cited (generated by the likelihood ratio of keywords). These keywords are highly susceptible to represent the overall topic of a cluster. Burstness is represented in each cluster with red tree-rings around nodes. [file Image_2.PDF]

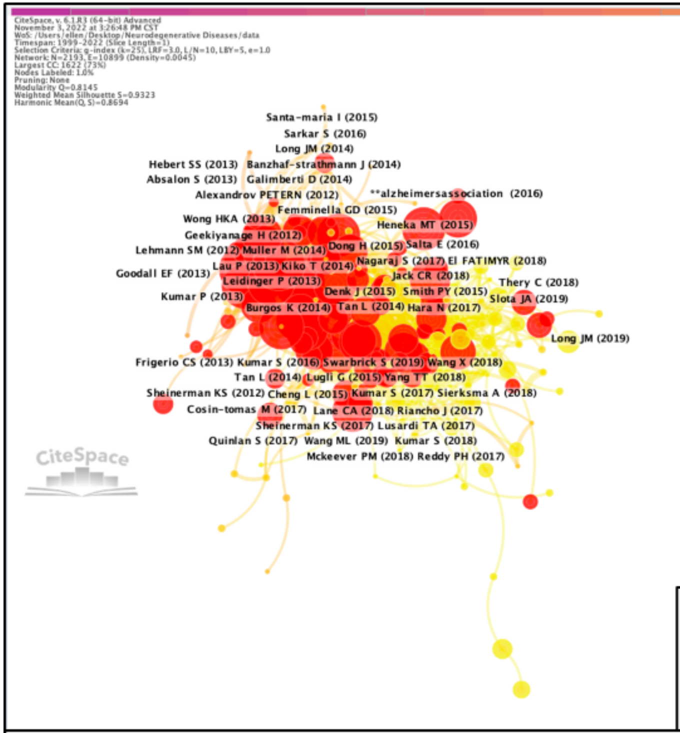

Cluster 0- 'alzheimers disease': alzheimers disease (118.66, 1.0E-4); dna methylation (53.3, 1.0E-4); epigenetics (53.07, 1.0E-4); mild cognitive impairment (51.11, 1.0E-4); biomarkers (40.45, 1.0E-4)

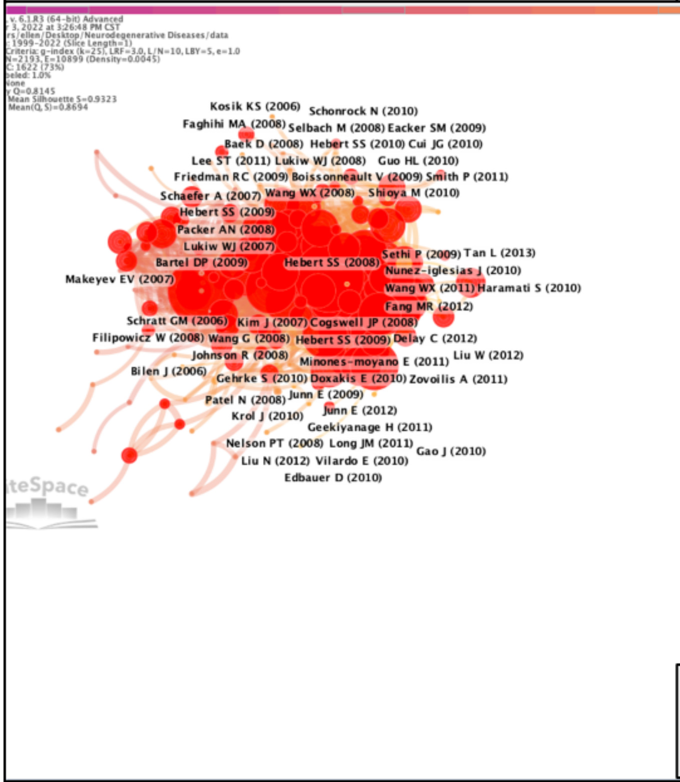

Cluster 1- 'microrna': microrna (66.18, 1.0E-4); dna methylation (26.01, 1.0E-4); neuron (19.22, 1.0E-4); neurodegeneration (14.92, 0.001); post-transcriptional (14.66, 0.001)

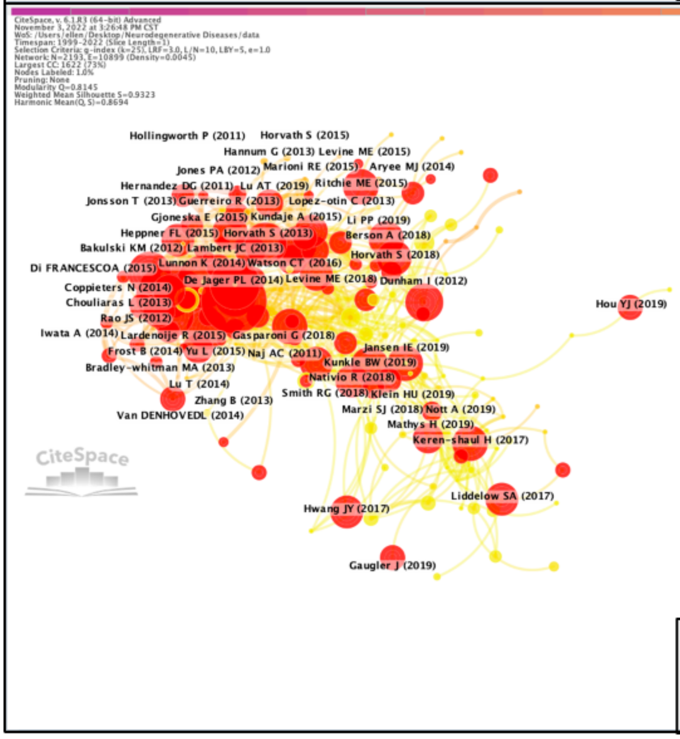

Cluster 2- 'dna methylation': dna methylation (156.86, 1.0E-4); epigenetics (82.17, 1.0E-4); microrna (70, 1.0E-4); aging (58.16, 1.0E-4); epigenetic clock (46.13, 1.0E-4)

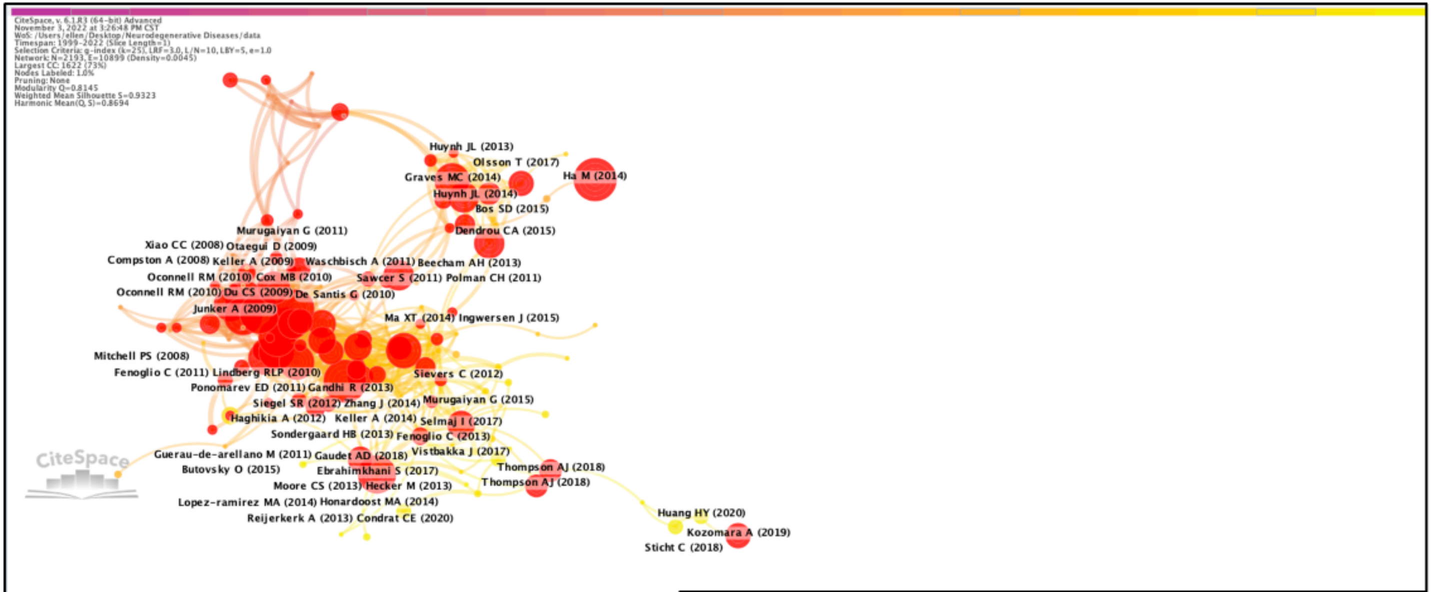

Cluster 3- 'multiple sclerosis': multiple sclerosis (393.17, 1.0E-4); alzheimers disease (89.62, 1.0E-4); autoimmunity (61.33, 1.0E-4); parkinsons disease (44.57, 1.0E-4); autoimmune disease (34.53, 1.0E-4)

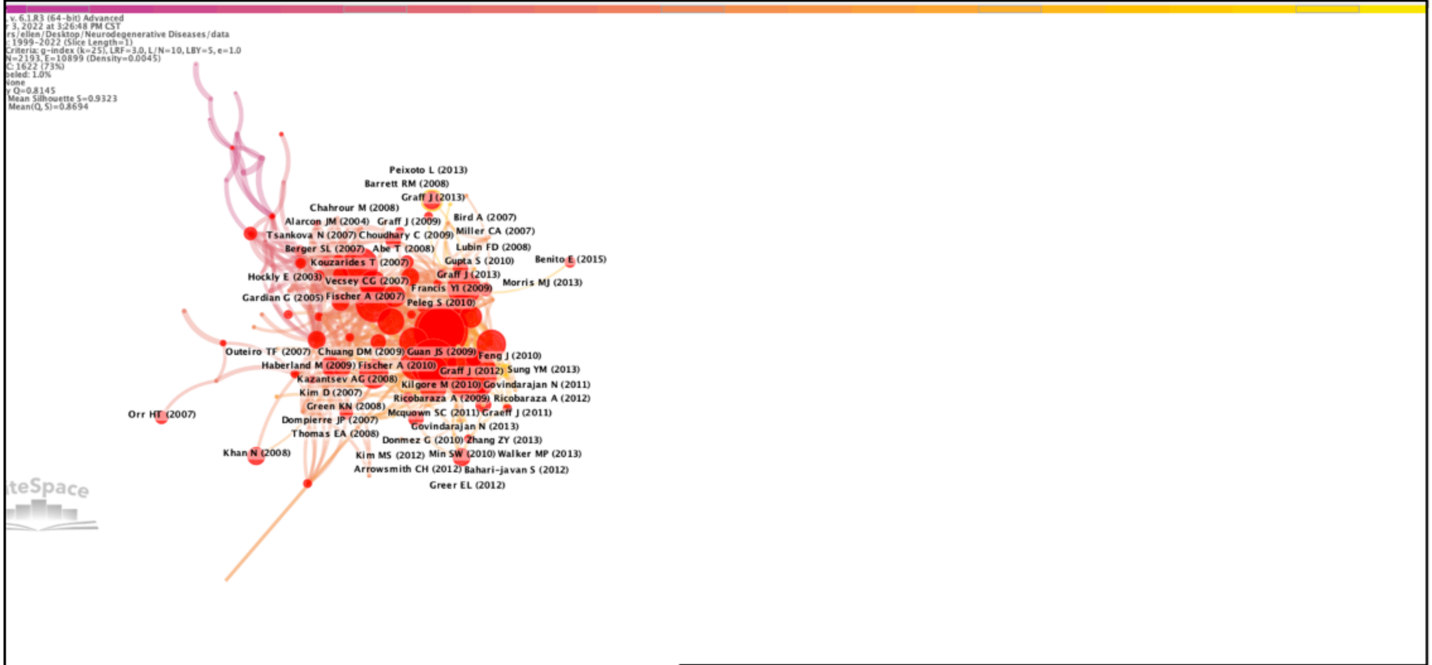

Cluster 4- 'histone acetylation': histone acetylation (89.03, 1.0E-4); histone deacetylase (72.95, 1.0E-4); epigenetics (53.34, 1.0E-4); microrna (50.22, 1.0E-4); hdac inhibitors (35.95, 1.0E-4)

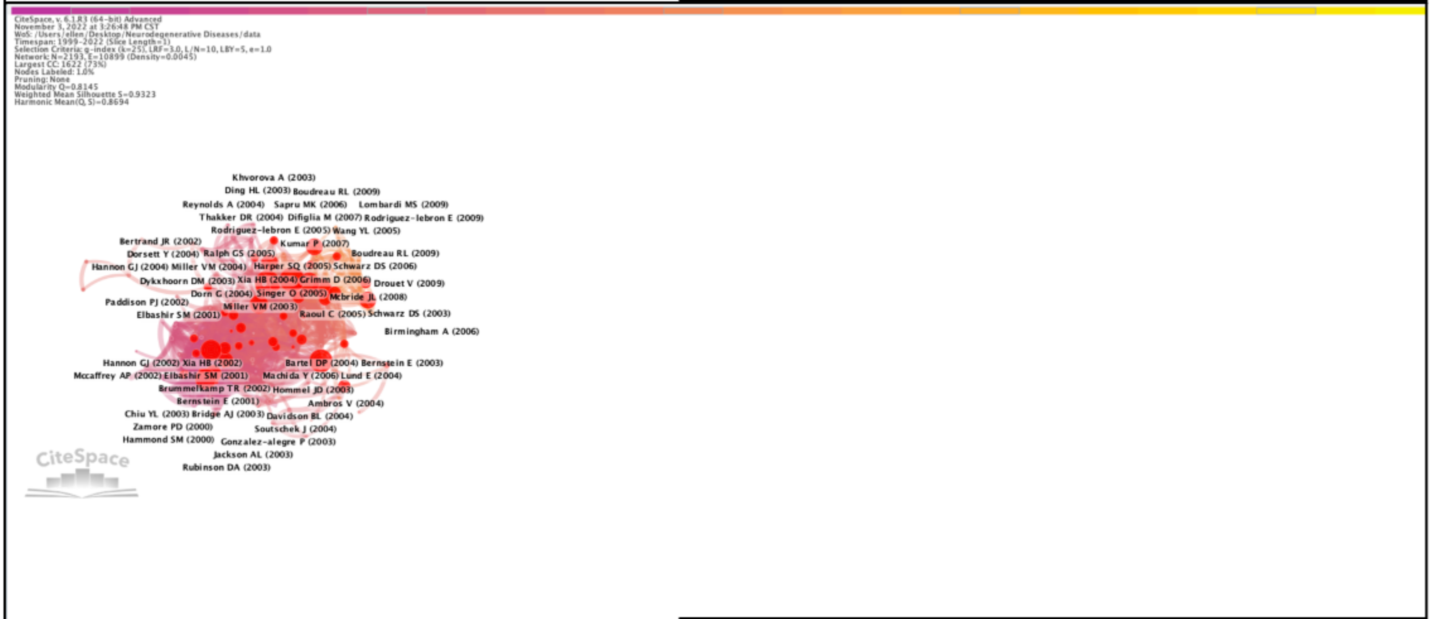

Cluster 5- 'rna interference': rna interference (89.94, 1.0E-4); gene therapy (74.28, 1.0E-4); sirna (65.69, 1.0E-4); rnai (55.73, 1.0E-4); gene silencing (38.66, 1.0E-4)

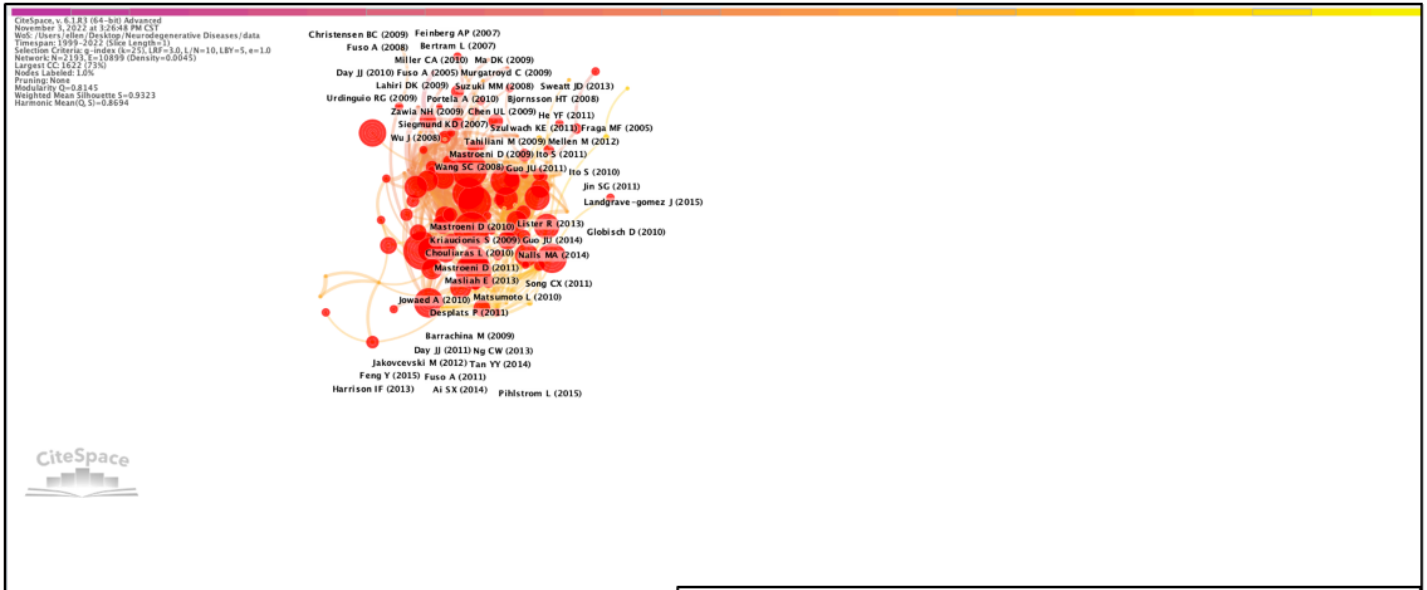

Cluster 6- 'epigenetics': dna methylation (112.61, 1.0E-4); epigenetics (86.78, 1.0E-4); microrna (48.61, 1.0E-4); 5-hydroxymethylcytosine (42.66, 1.0E-4); 5-methylcytosine (25.57, 1.0E-4)

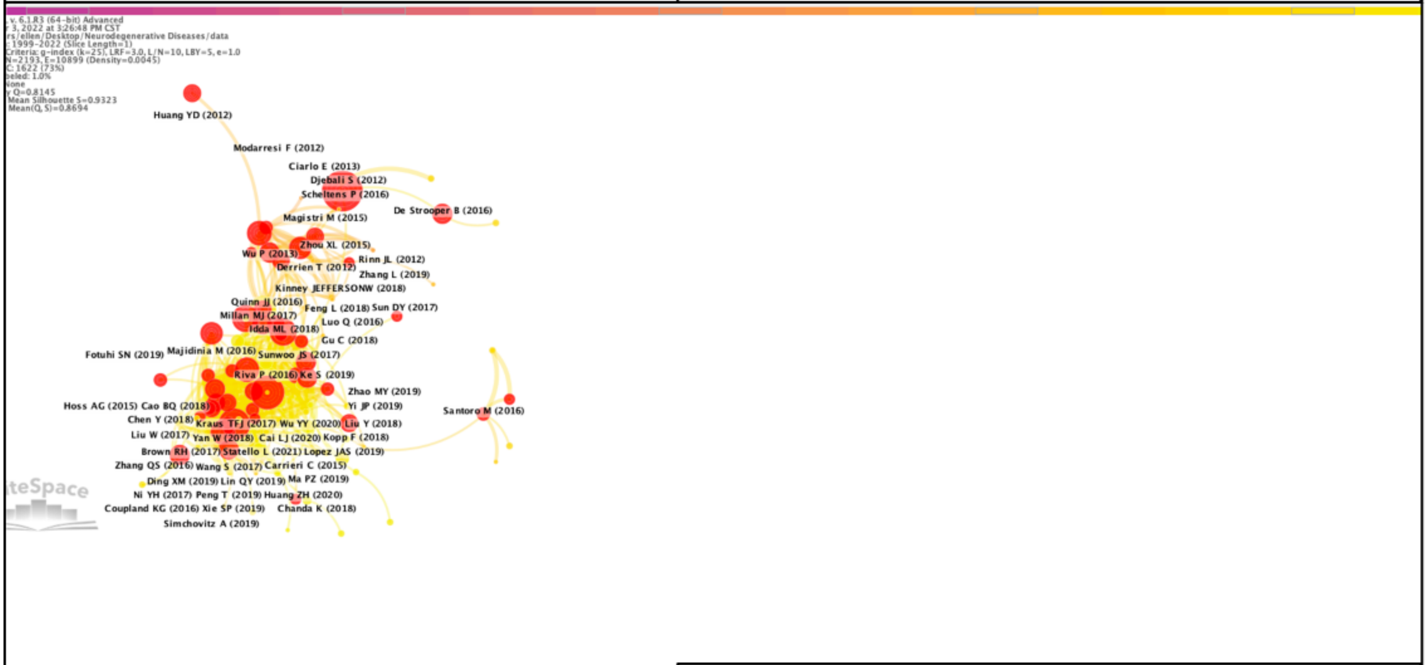

Cluster 7- 'lncrna': lncrna (80.56, 1.0E-4); neat1 (61.57, 1.0E-4); long non-coding rnas (57.85, 1.0E-4); long non-coding rna (46.93, 1.0E-4); lncrnas (29.45, 1.0E-4)

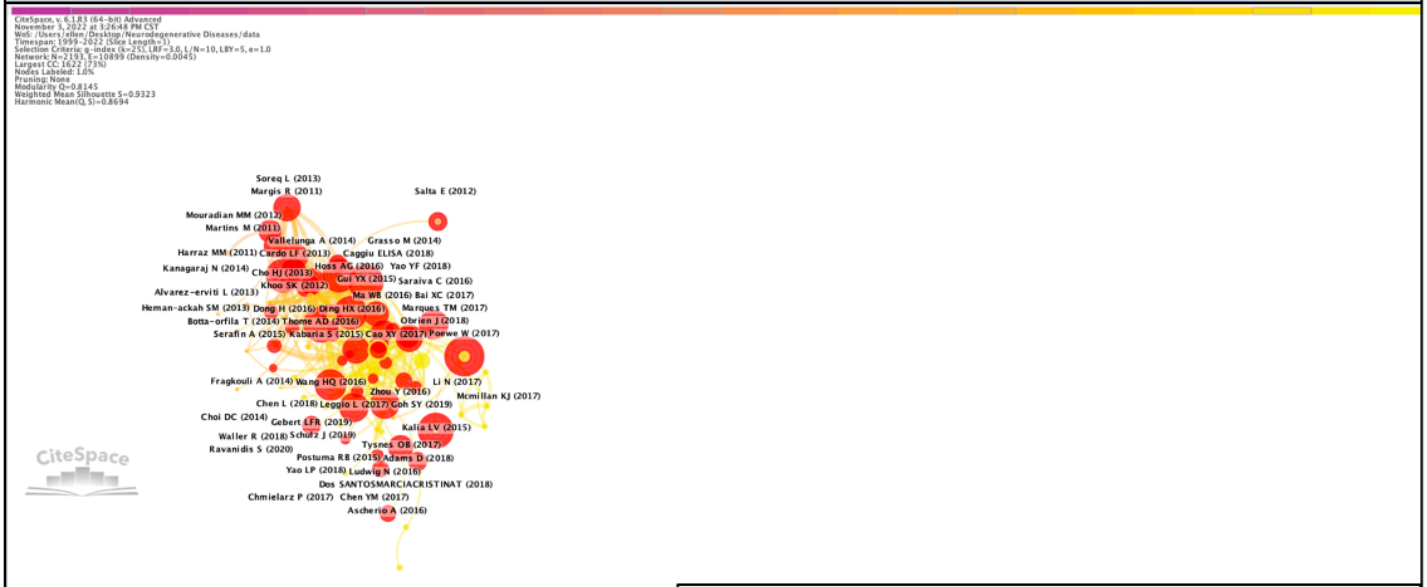

Cluster 8- 'parkinsons disease': parkinsons disease (211.52, 1.0E-4); alzheimers disease (50.95, 1.0E-4); dna methylation (42.96, 1.0E-4); alpha-synuclein (39.38, 1.0E-4); epigenetics (35.13, 1.0E-4)

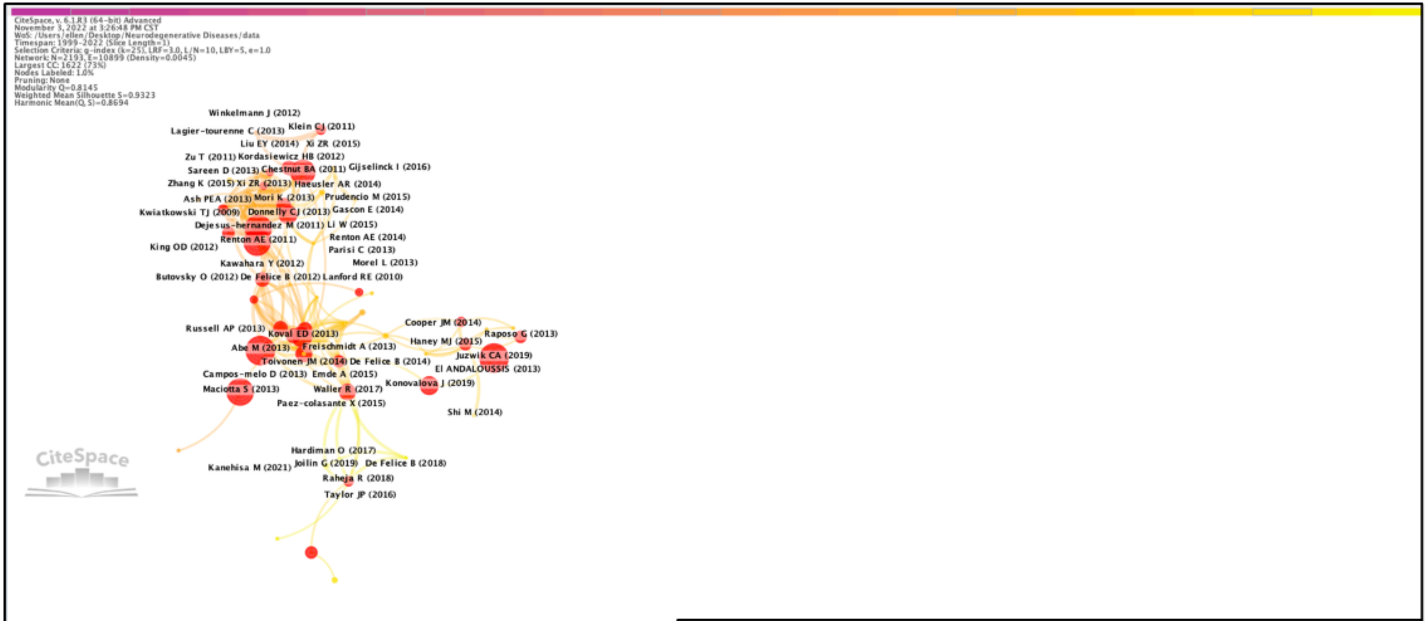

Cluster 9- 'amyotrophic lateral sclerosis': amyotrophic lateral sclerosis (92.32, 1.0E-4); als (79.9, 1.0E-4); c9orf72 (37.32, 1.0E-4); alzheimers disease (34.57, 1.0E-4); ftd (29.93, 1.0E-4)

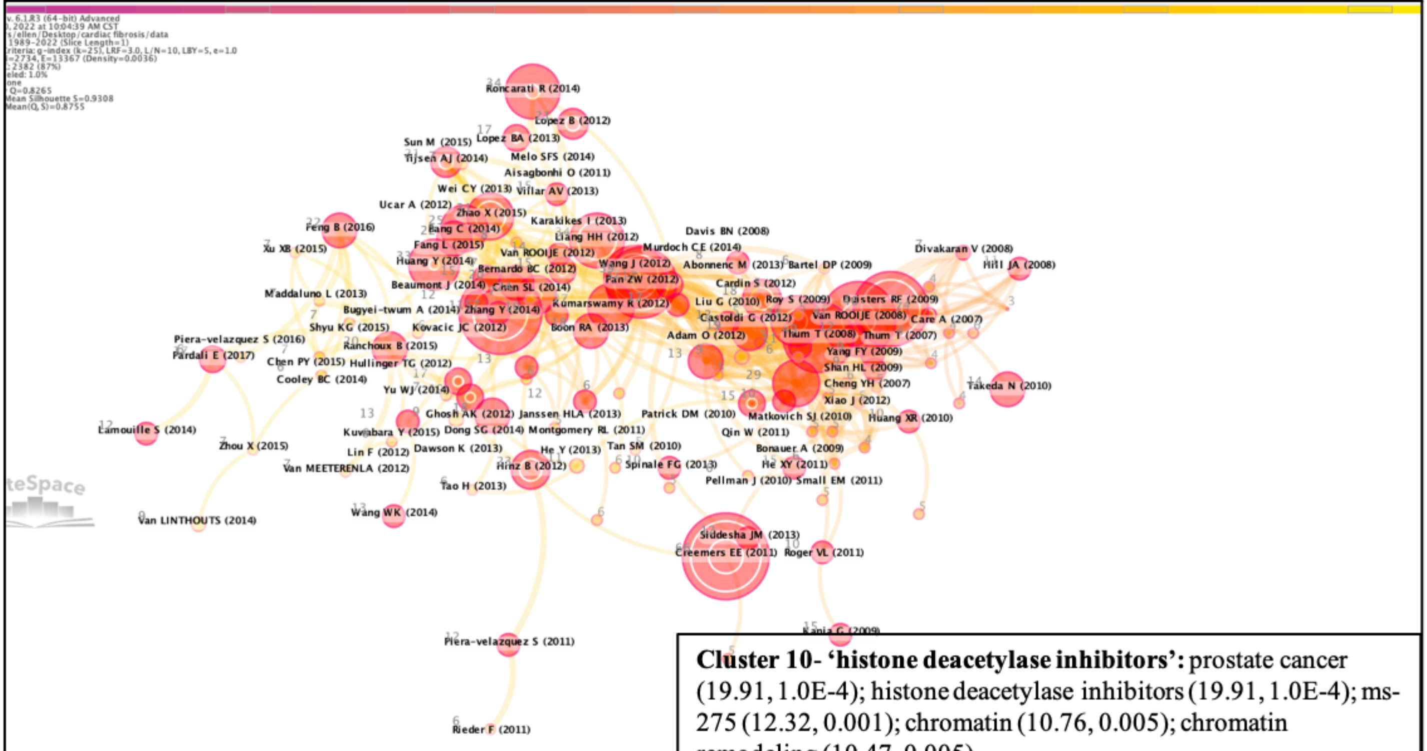

Cluster 10- 'histone deacetylase inhibitors': prostate cancer (19.91, 1.0E-4); histone deacetylase inhibitors (19.91, 1.0E-4); ms-275 (12.32, 0.001); chromatin (10.76, 0.005); chromatin remodeling (10.47, 0.005)

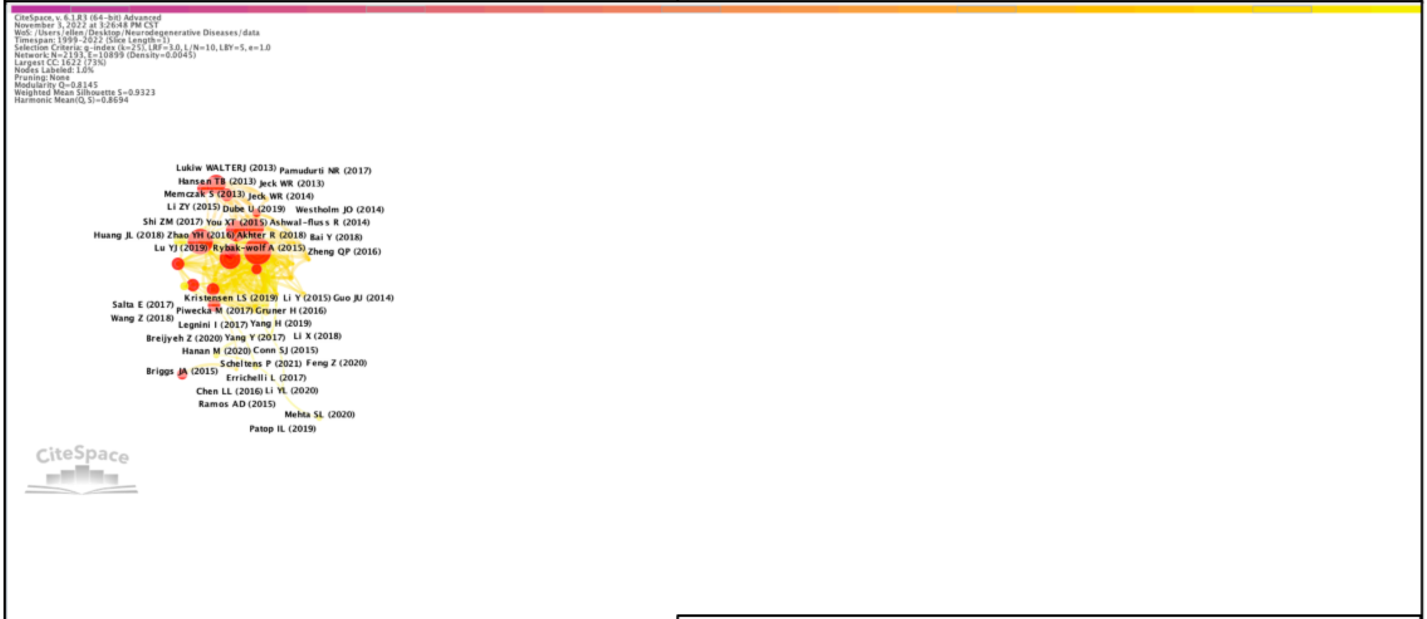

Cluster 12- 'circular rnas': circular rnas (58.1, 1.0E-4); circrnas (44.61, 1.0E-4); circular rna (38.16, 1.0E-4); circrna (29.47, 1.0E-4); dna methylation (17.69, 1.0E-4)

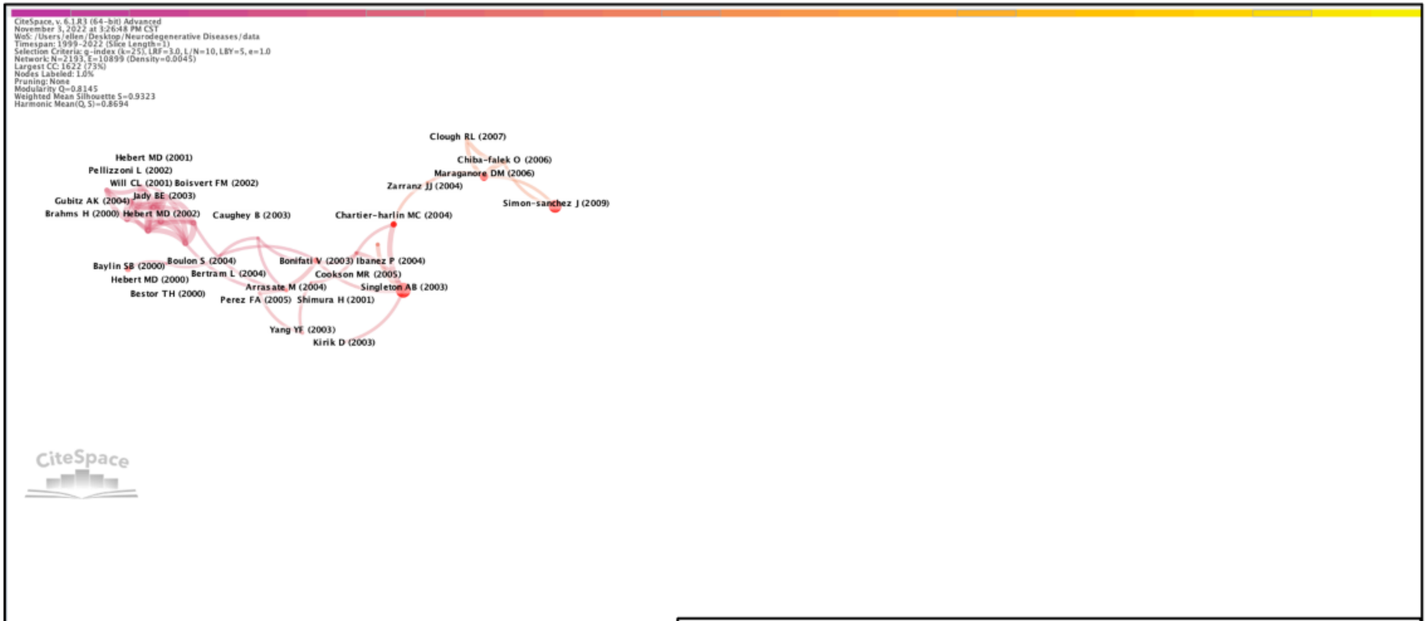

Cluster 14- 'alpha-synuclein': alpha-synuclein (17.19, 1.0E-4); neurotrophins (11.37, 0.001); pc12 (11.37, 0.001); unfolded protein response (11.37, 0.001); monoallelic (11.37, 0.001)

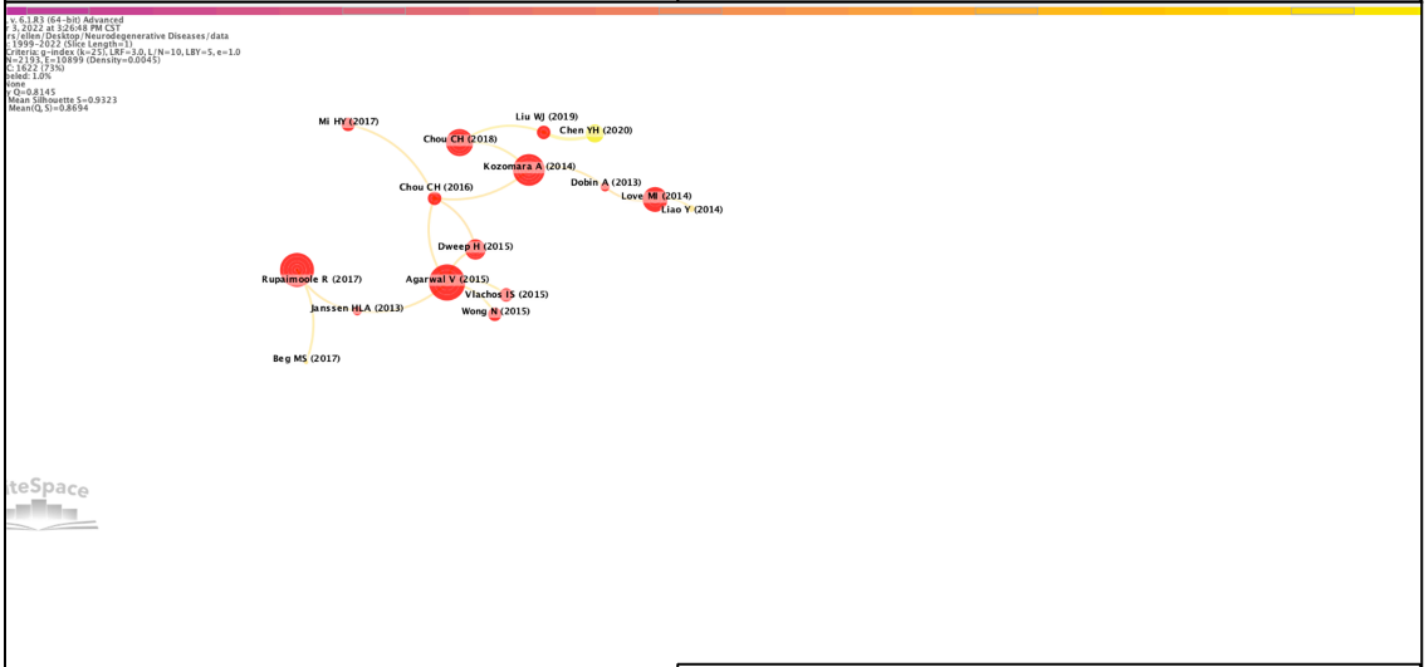

Cluster 18- 'network': dna methylation (22.56, 1.0E-4); epigenetics (17.98, 1.0E-4); network (12.76, 0.001); database (12.76, 0.001); interferon-beta (11.08, 0.001)

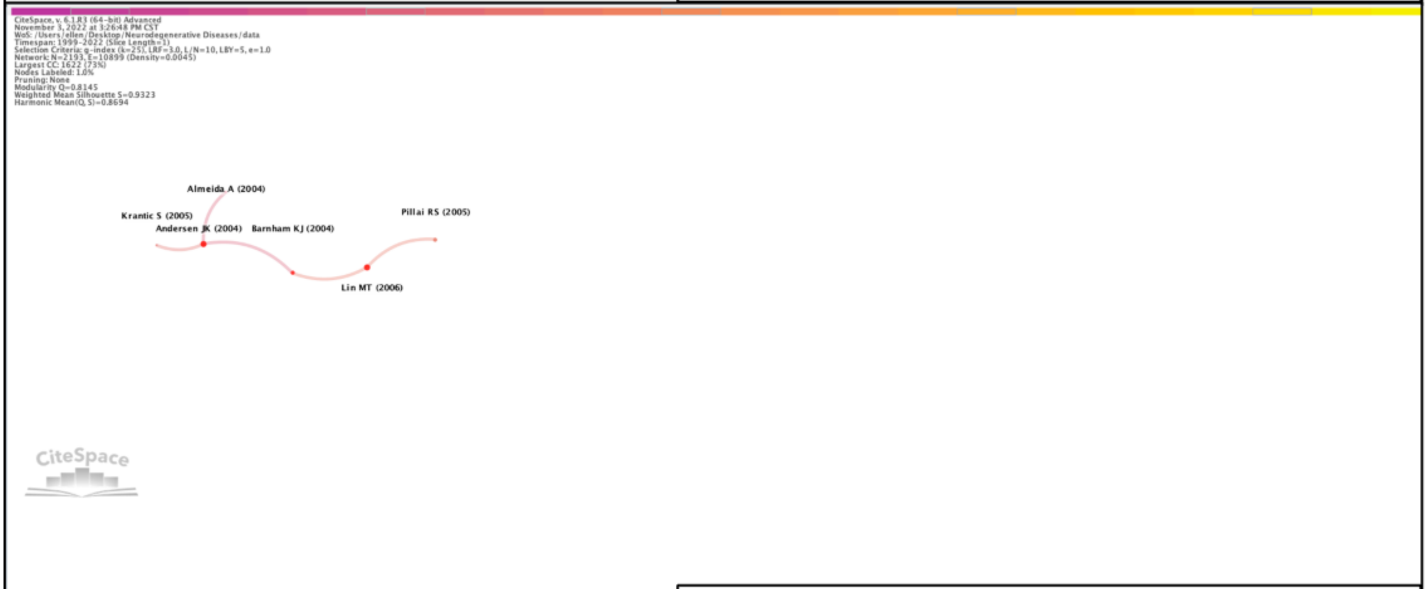

Cluster 20- 'mouse model': mouse model (12.31, 0.001); tetramethylpyrazine (12.31, 0.001); methylmalonic acidaemia (12.31, 0.001); flow cytometry (12.31, 0.001); codon usage (12.31, 0.001)

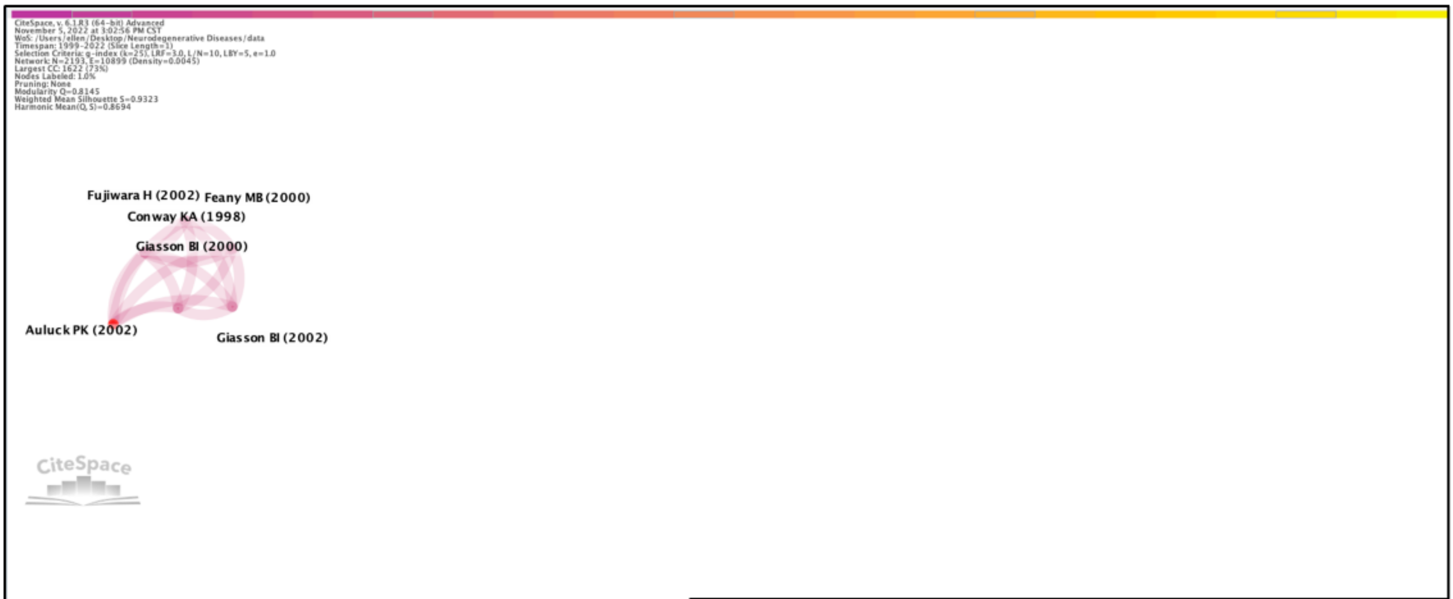

Cluster 21- ‘transgenic mice’: transgenic mice (37.12, 1.0E-4); heart rate variability (24.65, 1.0E-4); arrhythmias (13.53, 0.001); beta-adrenergic receptor kinase (12.28, 0.001); chaos (12.28, 0.001)

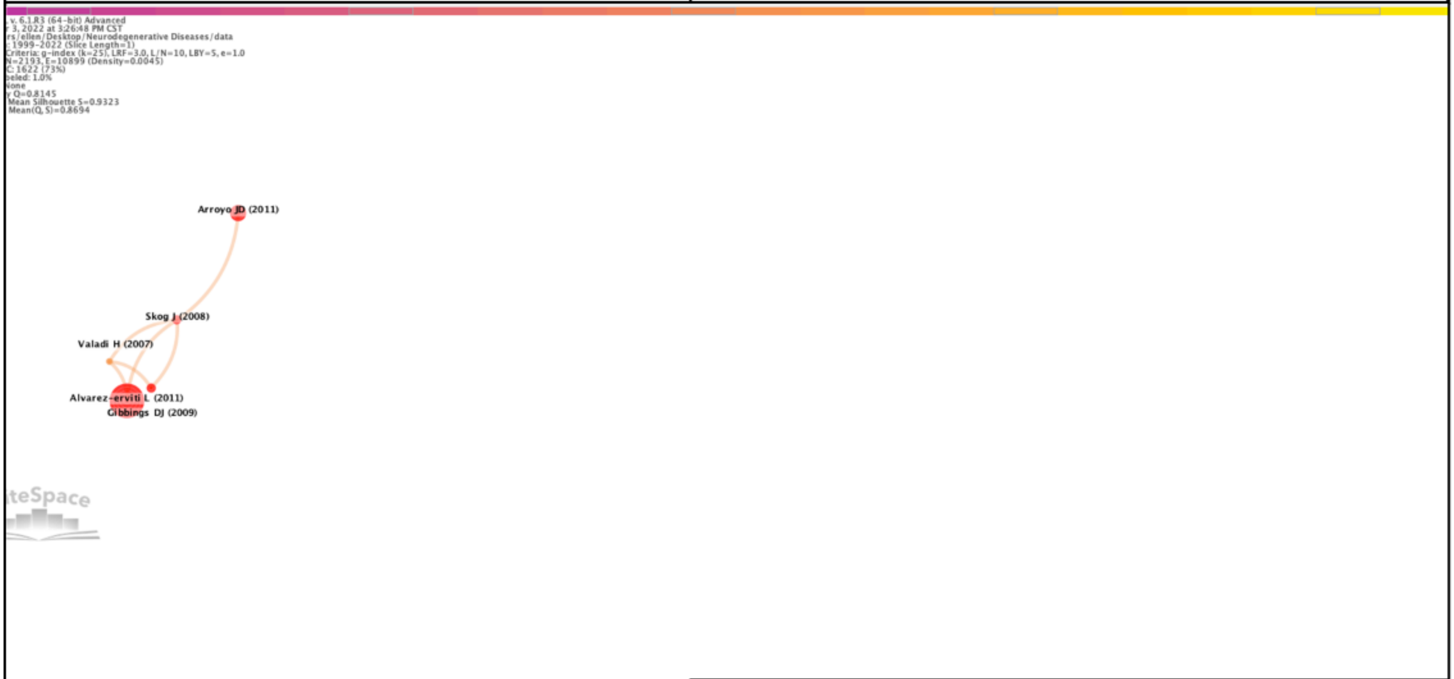

Cluster 23- : ‘microvesicles’: microvesicles (23.15, 1.0E-4); prions (18.4, 1.0E-4); exosomes (15.41, 1.0E-4); exosome (10.76, 0.005); centrifugation (9.19, 0.005)
