# Supplementary material for: Scoping review of epigenetics on neurodegenerative diseases: research frontiers and publication status
Source: Front Neurosci. 2024 Oct 9;18:1414603. doi: 10.3389/fnins.2024.1414603 (PMC11496254; doi:10.3389/fnins.2024.1414603)
Supplement: Supplementary Figure 3 — Link walkthrough between clusters based on burstness dynamic for co-cited reference network (1999–2022). [file Image_3.PDF]

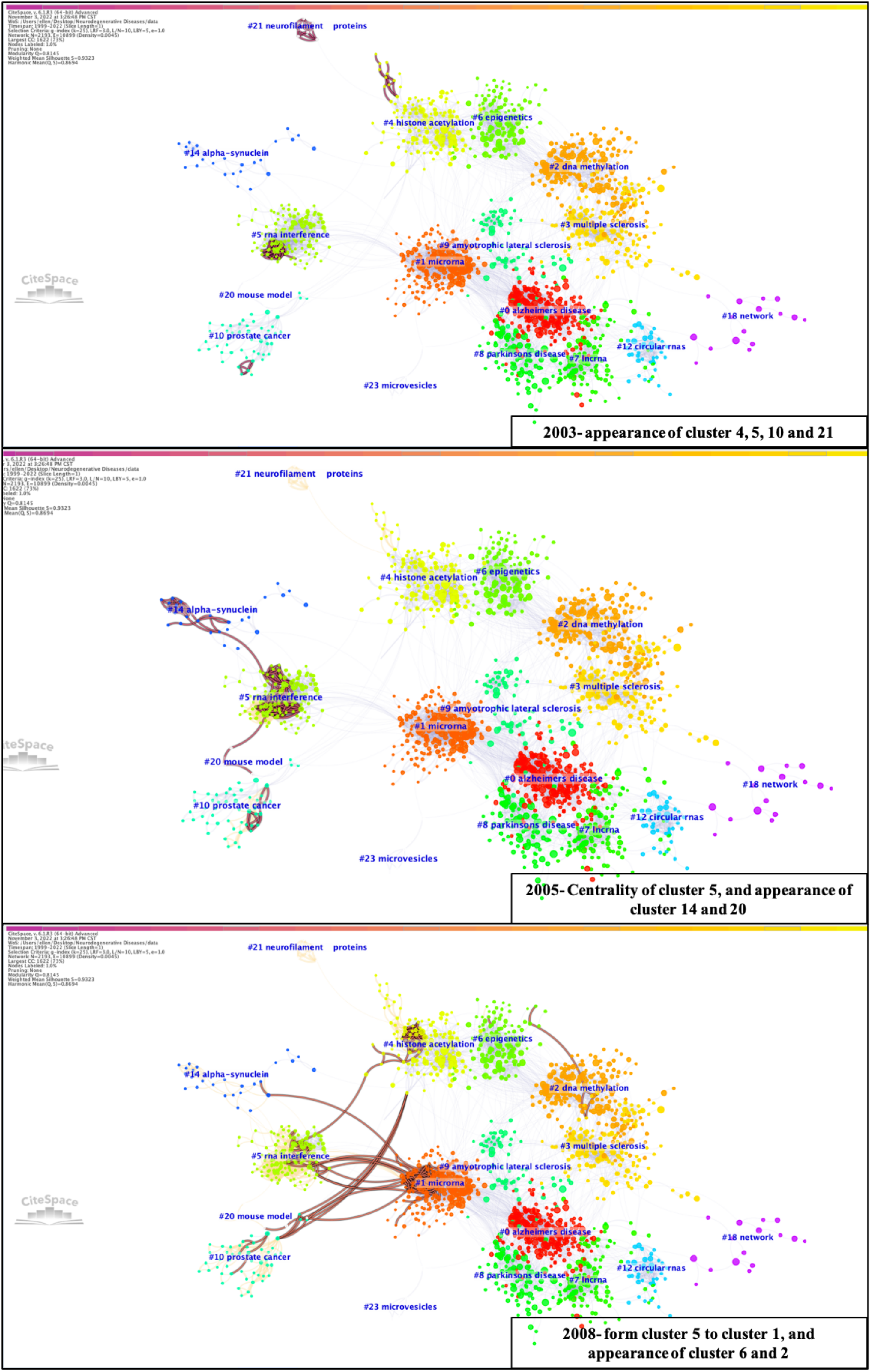
CiteSpace, v. 6.1.R3 (64-bit) Advanced
November 3, 2022 at 3:26:48 PM CST
WoS: /Users/ellen/Desktop/Neurodegenerative Diseases/data
Timespan: 1999-2022 (Slice Length=1)
Selection Criteria: g-index (k=25), LRF=3.0, L/N=10, LBY=5, e=1.0
Network: N=2193, E=10899 (Density=0.0045)
Largest CC: 1622 (73%)
Nodes Labeled: 1.0%
Pruning: None
Modularity Q=0.8145
Weighted Mean Silhouette S=0.9323
Harmonic Mean(Q, S)=0.8694
CiteSpace
#21 neurofilament proteins
#4 histone acetylation
#6 epigenetics
#14 alpha-synuclein
#2 dna methylation
#3 multiple sclerosis
#5 rna interference
#9 amyotrophic lateral sclerosis
#1 microrna
#20 mouse model
#0 alzheimers disease
#18 network
#10 prostate cancer
#12 circular rnas
#8 parkinsons disease
#7 lncrna
#23 microvesicles
2003- appearance of cluster 4, 5, 10 and 21
2005- Centrality of cluster 5, and appearance of cluster 14 and 20
2008- form cluster 5 to cluster 1, and appearance of cluster 6 and 2

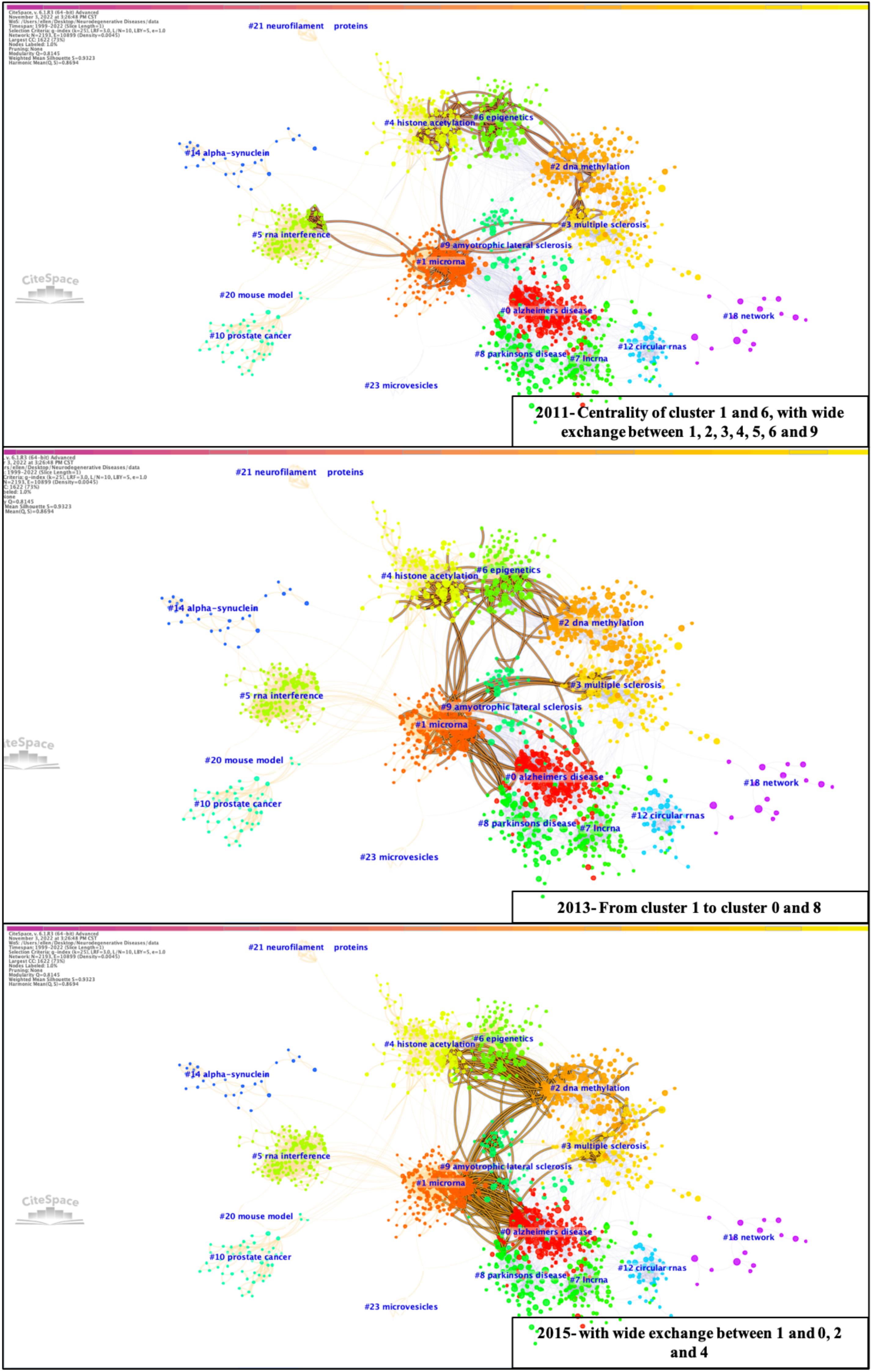
#21 neurofilament proteins
#4 histone acetylation
#6 epigenetics
#14 alpha-synuclein
#2 dna methylation
#5 rna interference
#3 multiple sclerosis
#9 amyotrophic lateral sclerosis
#1 microrna
CiteSpace
#20 mouse model
#0 alzheimers disease
#18 network
#10 prostate cancer
#12 circular rnas
#8 parkinsons disease
#7 lncrna
#23 microvesicles
2011- Centrality of cluster 1 and 6, with wide exchange between 1, 2, 3, 4, 5, 6 and 9
2013- From cluster 1 to cluster 0 and 8
2015- with wide exchange between 1 and 0, 2 and 4

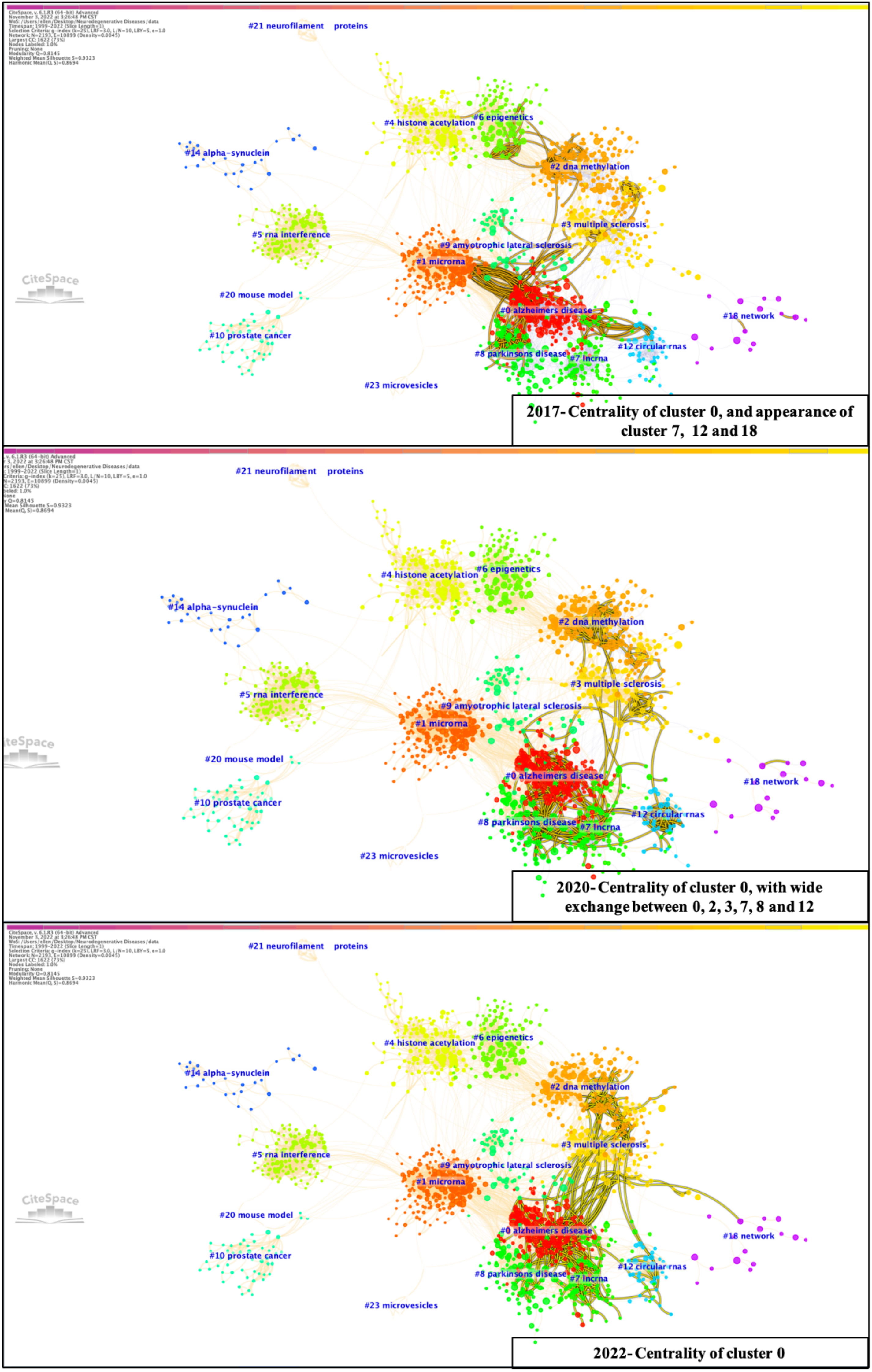

#21 neurofilament proteins
#4 histone acetylation
#6 epigenetics
#14 alpha-synuclein
#2 dna methylation
#5 rna interference
#3 multiple sclerosis
#9 amyotrophic lateral sclerosis
#1 microrna
CiteSpace
#20 mouse model
#0 alzheimers disease
#18 network
#10 prostate cancer
#12 circular rnas
#8 parkinsons disease
#7 lncrna
#23 microvesicles
2017- Centrality of cluster 0, and appearance of cluster 7, 12 and 18
#21 neurofilament proteins
#4 histone acetylation
#6 epigenetics
#14 alpha-synuclein
#2 dna methylation
#5 rna interference
#3 multiple sclerosis
#9 amyotrophic lateral sclerosis
#1 microrna
#20 mouse model
#0 alzheimers disease
#18 network
#10 prostate cancer
#12 circular rnas
#8 parkinsons disease
#7 lncrna
#23 microvesicles
2020- Centrality of cluster 0, with wide exchange between 0, 2, 3, 7, 8 and 12
#21 neurofilament proteins
#4 histone acetylation
#6 epigenetics
#14 alpha-synuclein
#2 dna methylation
#5 rna interference
#3 multiple sclerosis
#9 amyotrophic lateral sclerosis
#1 microrna
CiteSpace
#20 mouse model
#0 alzheimers disease
#18 network
#10 prostate cancer
#12 circular rnas
#8 parkinsons disease
#7 lncrna
#23 microvesicles
2022- Centrality of cluster 0
