# Supplementary material for: Scoping review of epigenetics on neurodegenerative diseases: research frontiers and publication status
Source: Front Neurosci. 2024 Oct 9;18:1414603. doi: 10.3389/fnins.2024.1414603 (PMC11496254; doi:10.3389/fnins.2024.1414603)
Supplement: Supplementary Figure 10 — Overlay visualization of most cited journals for the last 5 years (A), and most co-cited journals which published the most articles these last 30 years (B). Figure A is obtained with CiteSpace and Figure B with VOSviewer. (A) 13 clusters are identified. (B) Weighted on documents, Minimum number of citations of a journal = 150, 892 meet the thresholds, which are identified with 7 clusters. [file Image_10.PDF]

A

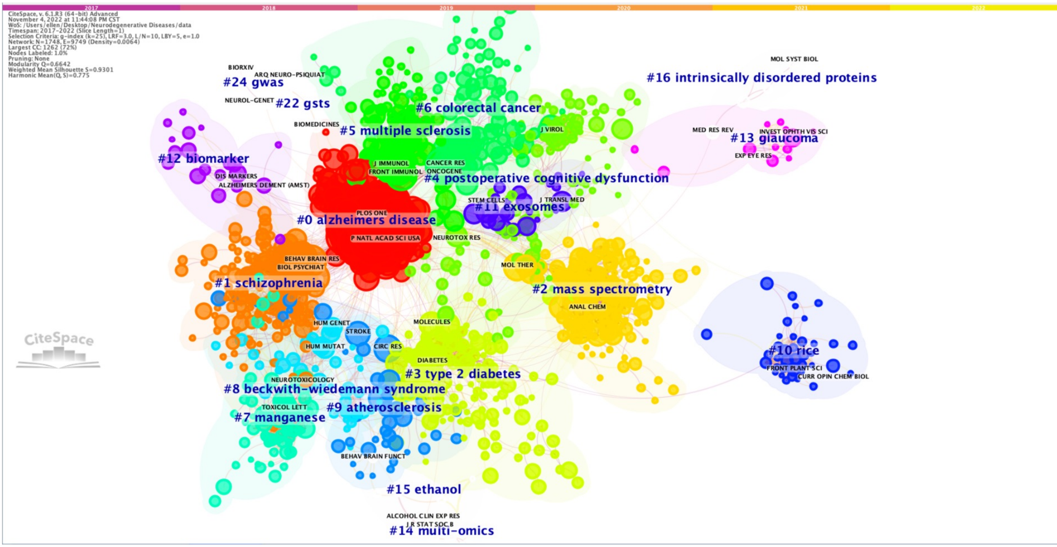

#24 gwas
#22 gsts
#12 biomarker
#6 colorectal cancer
#5 multiple sclerosis
#16 intrinsically disordered proteins
#13 glaucoma
#4 postoperative cognitive dysfunction
#11 exosomes
#0 alzheimers disease
#1 schizophrenia
#2 mass spectrometry
#10 rice
#3 type 2 diabetes
#8 beckwith-wiedemann syndrome
#9 atherosclerosis
#7 manganese
#15 ethanol
#14 multi-omics
BIORXIV
ARQ NEURO-PSIQUIAT
NEUROL-GENET
BIOMEDICINES
DIS MARKERS
ALZHEIMERS DEMENT (AMST)
J IMMUNOL
FRONT IMMUNOL
CANCER RES
ONCOGENE
J VIROL
MOL SYST BIOL
MED RES REV
INVEST OPHTH VIS SCI
EXP EYE RES
STEM CELLS
J TRANSL MED
PLOS ONE
P NATL ACAD SCI USA
NEUROTOX RES
BEHAV BRAIN RES
BIOL PSYCHIAT
MOL THER
ANAL CHEM
HUM GENET
STROKE
HUM MUTAT
CIRC RES
MOLECULES
DIABETES
FRONT PLANT SCI
CURR OPIN CHEM BIOL
NEUROTOXICOLOGY
TOXICOL LETT
BEHAV BRAIN FUNCT
ALCOHOL CLIN EXP RES
J R STAT SOC B
CiteSpace

B

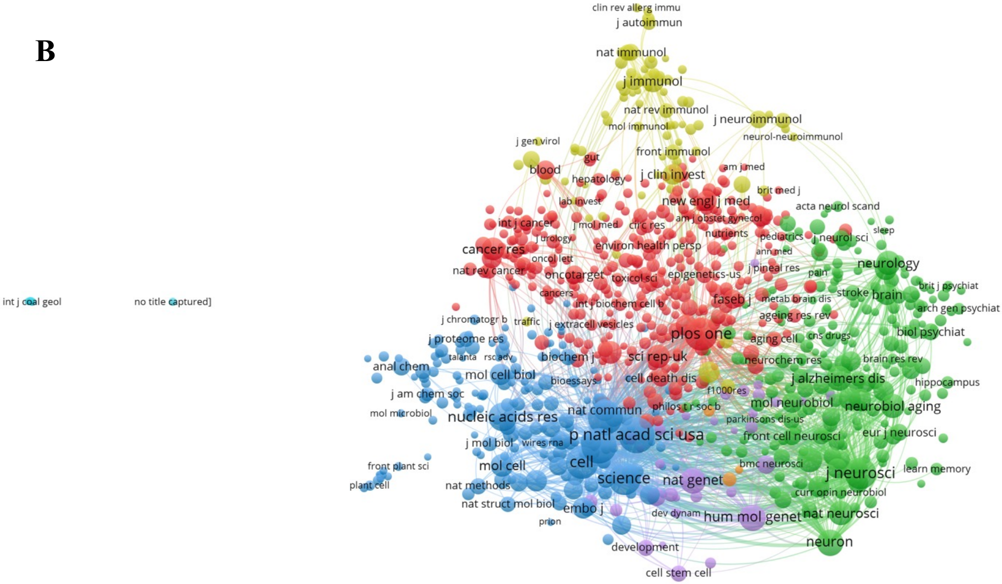

clin rev allerg immu
j autoimmun
nat immunol
j immunol
nat rev immunol
mol immunol
j neuroimmunol
neurol-neuroimmunol
j gen virol
gut
front immunol
blood
hepatology
j clin invest
am j med
brit med j
lab invest
new engl j med
acta neurol scand
int j cancer
j mol med
circ res
am j obstet gynecol
j urology
nutrients
pediatrics
j neurol sci
sleep
environ health persp
ann med
cancer res
oncol lett
neurology
nat rev cancer
oncotarget
toxicol sci
epigenetics-us
j pineal res
pain
brit j psychiat
cancers
stroke
brain
arch gen psychiat
int j biochem cell b
faseb j
metab brain dis
ageing res rev
j chromatogr b
traffic
j extracell vesicles
plos one
aging cell
cns drugs
biol psychiat
j proteome res
talanta
rsc adv
biochem j
sci rep-uk
neurochem res
brain res rev
anal chem
mol cell biol
bioessays
cell death dis
j alzheimers dis
hippocampus
j am chem soc
f1000res
mol neurobiol
neurobiol aging
int j coal geol
no title captured]
mol microbiol
nucleic acids res
nat commun
philos t r soc b
parkinsons dis-us
j mol biol
wires rna
p natl acad sci usa
front cell neurosci
eur j neurosci
bmc neurosci
front plant sci
cell
mol cell
science
nat genet
j neurosci
learn memory
plant cell
nat methods
curr opin neurobiol
nat struct mol biol
embo j
dev dynam
hum mol genet
nat neurosci
prion
development
neuron
cell stem cell
