# Supplementary material for: Scoping review of epigenetics on neurodegenerative diseases: research frontiers and publication status
Source: Front Neurosci. 2024 Oct 9;18:1414603. doi: 10.3389/fnins.2024.1414603 (PMC11496254; doi:10.3389/fnins.2024.1414603)
Supplement: Supplementary Figure 12 — Visualization of the author co-citation network (A), with corresponding clusters (B) and time map (C) (2017–2022). [file Image_12.PDF]

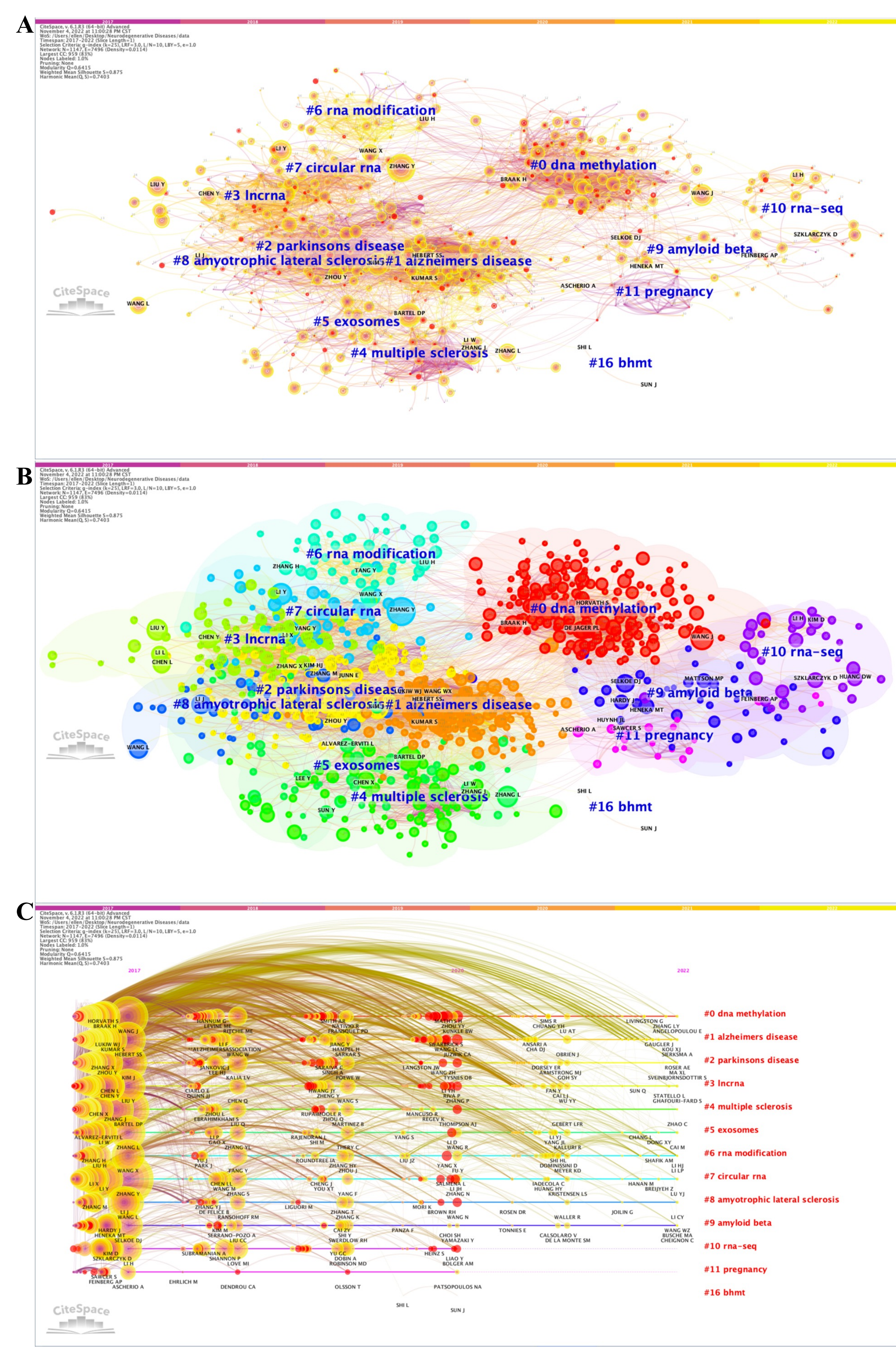
A
2017
2018
2019
2020
2021
2022
CiteSpace, v. 6.1.R3 (64-bit) Advanced
November 4, 2022 at 11:00:28 PM CST
WoS: /Users/ellen/Desktop/Neurodegenerative Diseases/data
Timespan: 2017-2022 (Slice Length=1)
Selection Criteria: g-index (k=25), LRF=3.0, L/N=10, LBY=5, e=1.0
Network: N=1147, E=7496 (Density=0.0114)
Largest CC: 959 (83%)
Nodes Labeled: 1.0%
Pruning: None
Modularity Q=0.6415
Weighted Mean Silhouette S=0.875
Harmonic Mean(Q, S)=0.7403
#6 rna modification
#7 circular rna
#0 dna methylation
#3 lncrna
#10 rna-seq
#2 parkinsons disease
#9 amyloid beta
#8 amyotrophic lateral sclerosis
#1 alzheimers disease
#11 pregnancy
#5 exosomes
#4 multiple sclerosis
#16 bhmt
CiteSpace
B
C
#0 dna methylation
#1 alzheimers disease
#2 parkinsons disease
#3 lncrna
#4 multiple sclerosis
#5 exosomes
#6 rna modification
#7 circular rna
#8 amyotrophic lateral sclerosis
#9 amyloid beta
#10 rna-seq
#11 pregnancy
#16 bhmt
