# Supplementary material for: Scoping review of epigenetics on neurodegenerative diseases: research frontiers and publication status
Source: Front Neurosci. 2024 Oct 9;18:1414603. doi: 10.3389/fnins.2024.1414603 (PMC11496254; doi:10.3389/fnins.2024.1414603)
Supplement: Supplementary Figure 9 — Top 10 growth source (1999–2022 and 2017–2022). [file Image_9.PDF]

A

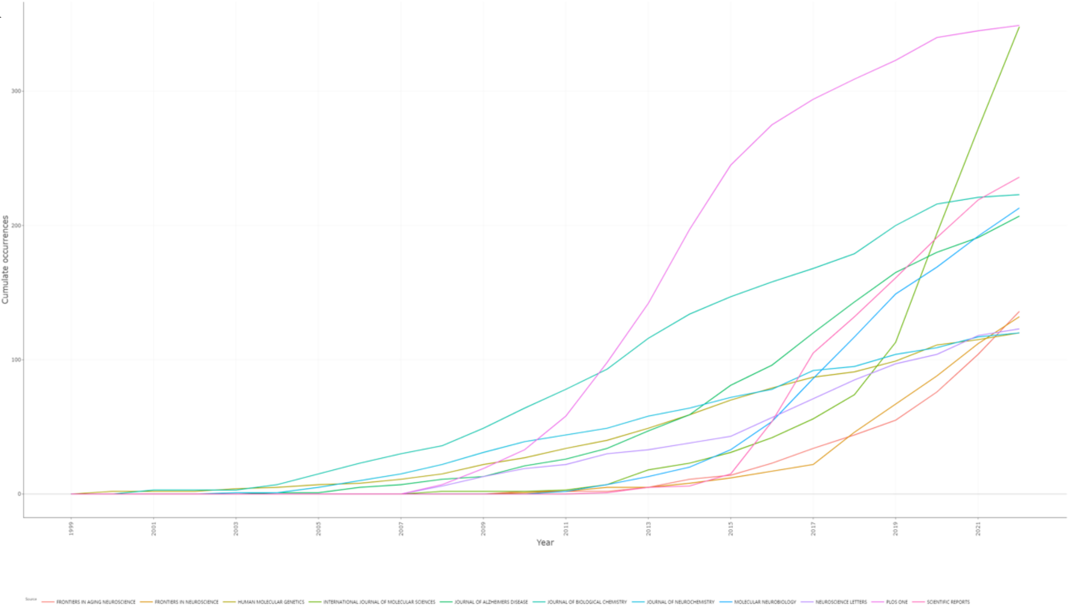
Cumulate occurrences
300
200
100
0
1999
2001
2003
2005
2007
2009
2011
2013
2015
2017
2019
2021
Year
Source
FRONTIERS IN AGING NEUROSCIENCE
FRONTIERS IN NEUROSCIENCE
HUMAN MOLECULAR GENETICS
INTERNATIONAL JOURNAL OF MOLECULAR SCIENCES
JOURNAL OF ALZHEIMERS DISEASE
JOURNAL OF BIOLOGICAL CHEMISTRY
JOURNAL OF NEUROCHEMISTRY
MOLECULAR NEUROBIOLOGY
NEUROSCIENCE LETTERS
PLOS ONE
SCIENTIFIC REPORTS

B

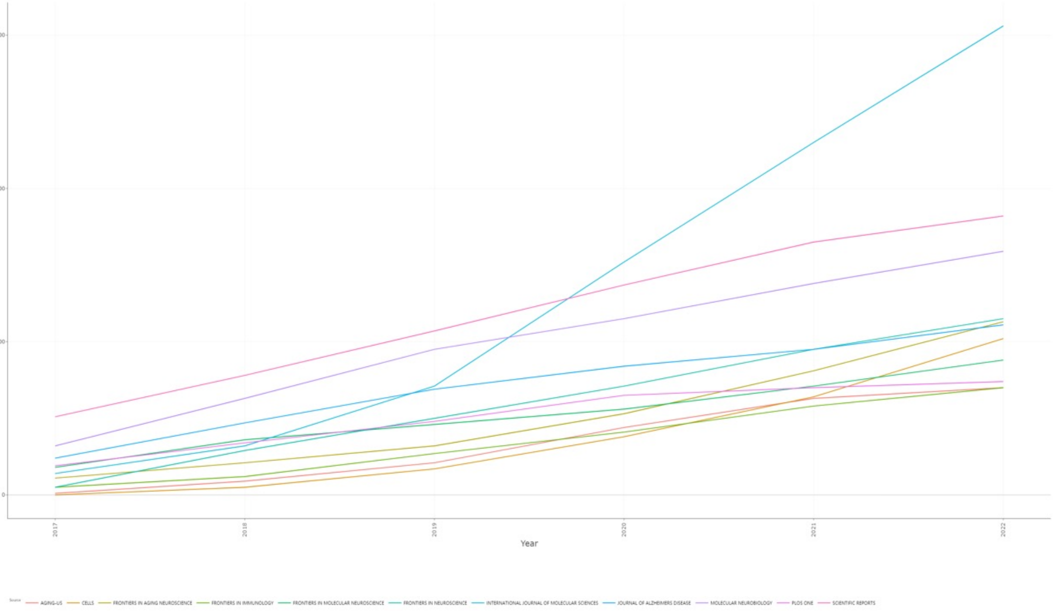
Cumulate occurrences
300
200
100
0
2017
2018
2019
2020
2021
2022
Year
Source
AGING-US
CELLS
FRONTIERS IN AGING NEUROSCIENCE
FRONTIERS IN IMMUNOLOGY
FRONTIERS IN MOLECULAR NEUROSCIENCE
FRONTIERS IN NEUROSCIENCE
INTERNATIONAL JOURNAL OF MOLECULAR SCIENCES
JOURNAL OF ALZHEIMERS DISEASE
MOLECULAR NEUROBIOLOGY
PLOS ONE
SCIENTIFIC REPORTS
