# Supplementary material for: Scoping review of epigenetics on neurodegenerative diseases: research frontiers and publication status
Source: Front Neurosci. 2024 Oct 9;18:1414603. doi: 10.3389/fnins.2024.1414603 (PMC11496254; doi:10.3389/fnins.2024.1414603)
Supplement: Supplementary Figure 11 — Co-authorship network obtained with VOSviewer. 25 clusters are identified comprising 489 different authors. Each cluster is identified with a different color. Minimum number of documents of an author = 8. [file Image_11.PDF]

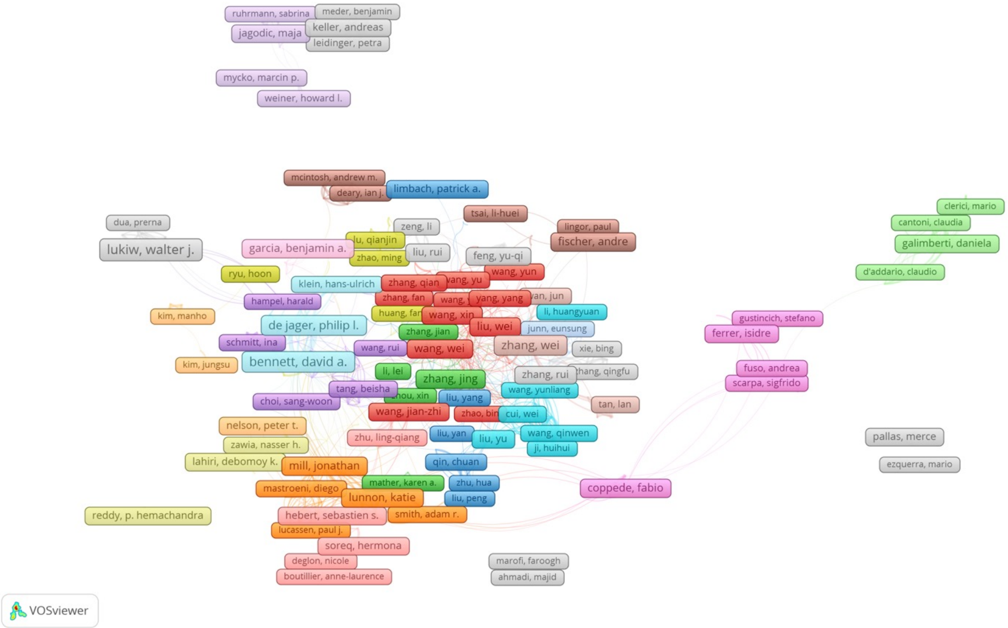
ruhrmann, sabrina
meder, benjamin
jagodic, maja
keller, andreas
leidinger, petra
mycko, marcin p.
weiner, howard l.
mcintosh, andrew m.
limbach, patrick a.
dua, prerna
tsai, li-huei
zeng, li
lingor, paul
lukiw, walter j.
garcia, benjamin a.
fischer, andre
liu, rui
feng, yu-qi
zhao, ming
ryu, hoon
wang, yun
klein, hans-ulrich
zhang, qian
zhang, fan
yang, yang
hampel, harald
kim, manho
wang, xin
li, huangyuan
de jager, philip l.
liu, wei
junn, eunsung
zhang, jian
schmitt, ina
wang, rui
wang, wei
zhang, wei
xie, bing
kim, jungsu
bennett, david a.
li, lei
zhang, jing
zhang, rui
tang, beisha
wang, yunliang
choi, sang-woon
liu, yang
tan, lan
wang, jian-zhi
cui, wei
nelson, peter t.
zhu, ling-qiang
liu, yan
liu, yu
wang, qinwen
zawia, nasser h.
ji, huihui
lahiri, debomoy k.
mill, jonathan
qin, chuan
mastroeni, diego
mather, karen a.
zhu, hua
lunnon, katie
liu, peng
reddy, p. hemachandra
hebert, sebastien s.
smith, adam r.
lucassen, paul j.
soreq, hermona
deglon, nicole
boutillier, anne-laurence
marofi, faroogh
ahmadi, majid
clerici, mario
cantoni, claudia
galimberti, daniela
d'addario, claudio
gustincich, stefano
ferrer, isidre
fuso, andrea
scarpa, sigfrido
coppede, fabio
pallas, merce
ezquerra, mario
VOSviewer
